# Enzymatic Browning in Wheat Kernels Produces Symptom of Black Point Caused by *Bipolaris sorokiniana*

**DOI:** 10.3389/fmicb.2020.526266

**Published:** 2020-12-09

**Authors:** Qiaoyun Li, Kaige Xu, Siyu Wang, Mengyu Li, Yumei Jiang, Xiaolong Liang, Jishan Niu, Chenyang Wang

**Affiliations:** National Engineering Research Centre for Wheat/National Key Laboratory of Wheat and Maize Crop Science, Henan Agricultural University, Zhengzhou, China

**Keywords:** wheat, black point, enzymatic browning, metabolomics, spectroscopic properties, symptom production

## Abstract

To understand the blackening mechanism in black point diseased kernels, ultraviolet–visible light (UV–Vis) and Fourier-transform infrared (FT-IR) absorbance spectra of extracts made from the blackening parts of black point-affected (BP) kernels and the analogous part of black point-free (BPF) kernels were measured using susceptible wheat genotypes “PZSCL6” inoculated with *Bipolaris sorokiniana* (the dominant pathogen causing this disease). In addition, metabolite differences between BP and BPF kernels were identified by a method that combines gas chromatography-mass spectrometry (GC-MS) and liquid chromatography-high resolution mass spectrometry (LC-MS). Successively, symptoms of black point were produced *in vitro*. The results showed (i) the spectroscopic properties of the extracts from BP and BPF kernels were very similar, with an absorption peak at 235 nm and a small shoulder at 280–300 nm in both UV–Vis spectra and shared vibrations at 3400–3300, 2925 and 2852, 1512 and 1463, 1709, 1220, 600–860 cm^–1^ in FT-IR spectra that are consistent with similar bonding characteristics. In contrast, spectroscopic properties of extracts from wheat kernels were different from those of synthetic melanin and extracellular and intracellular melanin produced by *B. sorokiniana*. (ii) Levels of 156 metabolites in BP kernels were different from those in BPF kernels. Among those 156 metabolites, levels of phenolic acids (ferulic acid and p-coumaric acid), 11 phenolamides compounds, and four benzoxazinone derivatives were significantly higher in BP kernels than in BPF kernels. (iii) Symptom of black point could be produced *in vitro* in wheat kernels with supplement of phenol substrate (catechol) and H_2_O_2_. This result proved that blackening substance causing symptom of black point was produced by enzymatic browning in wheat kernels instead of by *B. sorokiniana*.

## Introduction

Black point, characterized by a dark discoloration of the embryo of kernels, is a serious disease in wheat growing regions throughout the world (Conner, 1989; Conner et al., 1992, 1996; Fernandez et al., 1994; Williamson, 1997; Mak et al., 2006; Moschini et al., 2006; Toklu et al., 2008; Xu et al., 2018; Li et al., 2020). It causes economic losses for farmers due to the reduction in the commercial grade of the wheat (Rees et al., 1984; Conner and Davidson, 1988) and decreases seed germination, inhibits seedling growth, and reduces grain yield (Conner et al., 1996; Li et al., 2014; El-Gremi et al., 2017). Furthermore, some fungal species that cause black point (e.g., *Bipolaris sorokiniana*, *Alternaria alternata*, and *Fusarium proliferatum*) may also produce toxic substances (Gauthier and Keller, 2013; Palacios et al., 2015; Masiello et al., 2020). In the Yellow and Huai wheat area of China, 62.5% of the 403 wheat genotypes were susceptible to black point during 2010–2012 (Li et al., 2014). Understanding the blackening mechanism of this disease is of great importance to studying the mechanism of resistance and control of this disease.

Current information about blackening mechanism of black point is incomplete and sometimes contradictory. Historically, it has been assumed that discoloration is the result of colonization by fungi, including *A. alternata*, *B. sorokiniana*, and *Fusarium* spp. (Bhowmik, 1969; Southwell et al., 1980; Rees et al., 1984; Conner et al., 1996; Kumar et al., 2002). However, other research on wheat and barley did not provide evidence supporting that symptoms of black point directly link to fungal colonization (Conner and Davidson, 1988; Basson et al., 1990; Ellis et al., 1996; Williamson, 1997). Some researchers have suggested that biochemical changes induced by pathogenic infection and environmental stress causes enzymatic browning within kernels, which results in black point symptoms (Williamson, 1997; Walker and Ferrar, 1998; Walker, 2011). Black point was reproduced *in vitro* when kernels were incubated in a phenol solution and then soaked in H_2_O_2_ solution (Williamson, 1997). However, there have been no reports that focus on the potential for diseased kernels to produce the blackening substance using metabolomics analysis and spectroscopic properties of the extracts from black point-affected (BP) and black point-free (BPF) kernels. Moreover, if the blackening substance causing symptom black point is related to the infection of fungi, whether it is produced by fungi or the result of biochemical changes within wheat kernels remains unclear.

In our previous study, we screened seven wheat genotypes that are highly susceptible to black point (Li et al., 2014) and identified eight fungal species, including *B. sorokiniana*, *A. alternata*, and *Fusarium equiseti*, that cause black point in the North China Plain (Xu et al., 2018), of which *B. sorokiniana* was the most virulent. Here we applied a metabolomics analysis to both the germ fraction (the main discolored part of the kernel) and the endosperm-bran fraction of BP and BPF kernels inoculated with *B. sorokiniana* using a method that combines gas chromatography-mass spectrometry (GC-MS) and liquid chromatography-high resolution mass spectrometry (LC-MS). Moreover, the spectroscopic properties of an extract from discolored area of diseased kernels were analyzed. Through comparing differential metabolites between BP and BPF kernels, and applying spectroscopic properties analysis to the blackening substance of wheat, melanin of *B. sorokiniana* and a melanin standard, the objectives of this study were to determine (i) whether the discoloration substance causing symptoms of black point is produced by wheat or by pathogen and (ii) whether the discoloration substance is produced by enzymatic browning.

## Experimental Procedures

### The Plant Material

Four wheat genotypes, of which two (“SP1777-1-2” and “Shannong4143”) were resistant and two (“PZSCL6” and “Aifeng66”) were susceptible to black point (Li et al., 2014) were used in this study. “PZSCL6,” used for all analysis, was planted in 12 rows that were 2 m long spaced 20 cm apart during 2016–2017 seasons in experimental field of Henan Agricultural University, Zhengzhou, Henan Province, China (longitude 113°42′E, latitude 34°44′N, elevation 111.3 m). The remaining three genotypes, used to produce symptom of black point *in vitro*, were planted in two rows. Fertilization, pest control, and weed management were conducted following Li et al. (2014).

### Inoculation With the Pathogen

The fungal isolate Ta-BP33, representing *B. sorokiniana* (Xu et al., 2018), was cultured on Potato Dextrose Agar (PDA) medium in 9-cm Petri dish and incubated for 10–12 days in a dark growth chamber at 25 ± 1°C. Conidial suspensions were prepared according to the procedure described by Mahto et al. (2011), and the inoculation was as previously reported (Li et al., 2019b). Briefly, at Zadoks growth stage GS 55 (Zadoks et al., 1974), 200 spikes of genotype “PZSCL6” were covered with sulfuric acid paper bags to prevent contamination, and then at GS 65 the spikes were inoculated with conidial suspensions using a hand sprayer until dripping off and covered with transparent plastic bags for 5 days to maintain humidity, after which the spikes were again covered with sulfuric acid paper bags until harvest.

When symptoms of black point were clearly visible at GS 87 (Hard dough), these 200 spikes inoculated with *B. sorokiniana* were collected and taken to the laboratory. BP and BPF kernels were selected quickly. Samples for metabolomics analysis were made from 50 BP and BPF kernels, each. Remaining kernels were used to extract blackening substance. Five biological replicates were used in metabolomics analysis, and four biological replicates were used for other analysis.

In metabolomics analysis, the germ, with adhering aleurone layer, was designated as the “germ fraction”; the remaining part of the kernel was designated as the “endosperm-bran fraction.” The abbreviations of the four sample groups are: GBP = germ fraction of BP, EBP = endosperm-bran fraction of BP, GBPF = germ fraction of BPF, and EBPF = endosperm-bran fraction of BPF.

### Extraction and Purification of Blackening Substance From Wheat Kernels

Blackening substance was extracted according to the basic procedure designed for isolating melanin pigments from tea with some adjustments (Sava et al., 2001). Kernels were soaked in water for 10 h, and then the coats of black/brown areas (around embryo end) were peeled off from BP kernels. Coats were also peeled off from the analogous part of BPF kernels. The peeled coats were washed with boiling water at a volume ratio of 1:20 (coats/water) for 15 min and then immersed in 0.1 M NaOH (1:20 g/ml). After 4 h incubation at 65°C (the black/brown on the coats from BP kernels has faded and is almost invisible with naked eye), the mixture was filtered and then centrifuged at 12,000 × *g* for 30 min. Next, the supernatant was acidified by the addition of 2 M HCl to pH 2.5, incubated at room temperature for 2 h, and centrifuged at 12,000 × *g* for 15 min to yield the blackening substance extracts.

Purification of the extracts was performed with acid hydrolysis, organic solvent treatment, and repeated precipitation as previously reported (Sava et al., 2001). The purified extracts were dried in a dryer for 24 h and stored at −20°C.

### Extraction of Melanin From *B. sorokiniana*

#### Intracellular Melanin (Bs-in)

Cultures of *B. sorokiniana* isolate Ta-BP33 were grown on PDA medium at 25°C in the dark for 15 days. Bs-in was extracted from the mycelia and purified by a method reported by Carzaniga et al. (2002). Briefly, mycelia were washed with deionized water, homogenized, and extracted with 1 M NaOH. After removal of the fungal residue by centrifugation, melanin was precipitated from the supernatant with 2 M HCl, washed with hexane, redissolved in NaOH and precipitated with acid, and finally washed extensively with deionized water. The purified melanin was dried in a dryer for 24 h, and stored at −20°C.

#### Extracellular Melanin (Bs-ex)

Inocula were maintained on PDA, then transferred into a 250 ml flask containing 100 ml potato dextrose medium (pH 10.0), and grown at 25°C for 12 days with continuous 150 r/min shaking. Bs-ex was extracted using a previously reported procedure (Wu et al., 2008). Culture medium was filtered to remove mycelia and insoluble particles. The pH value of the derived supernatant was adjusted to 2.5 with 2 M HCl. After a short incubation, the suspension was centrifuged (4000 *g*, 10 min) and the pellet was collected. The purification and storage of Bs-ex was the same as for Bs-in.

### Ultraviolet–Visible (UV) Spectroscopy

Extracts from BP and BPF kernels, intracellular and extracellular melanin from *B. sorokiniana*, and a melanin standard (Synthetic melanin, Sigma, M8631) were separately dissolved to a final concentration of 10 mg L^–1^ in phosphate buffer (pH 8.0). Each solution’s absorption spectrum (190–800 nm) was recorded using a UV spectrophotometer (AOE Instruments A590, China). Each sample was analyzed three times.

### Fourier-Transform Infrared (FT-IR) Analysis

The extraction samples from wheat kernels, melanin from *B. sorokiniana*, and synthetic melanin were mixed with KBr powder at a ratio of 1:100, and then pressed into 1 mm pellets for Fourier-transform infrared (FT-IR) measurement. FT-IR spectra (4000–400 cm^–1^) were determined using a FT-IR spectrophotometer (Nicolet 5700, ThermoNicolet, United States).

### Metabolomics Analysis

#### Metabolite Extraction, Sample Derivatization, and GC-MS Analysis

The sample (100 ± 1 mg) was homogenized in 500 μl pre-chilled methanol/water (4:1, v/v) using a TissueLyzer (JX-24, Jingxin, Shanghai) containing zirconia beads for 4 min at 40 Hz. The homogenates were placed at −20°C for 2 h prior to centrifugation at 16,000 × *g* (4°C) for 15 min. Extraction of the solid residue was repeated with 400 μl of pre-chilled methanol/water (4:1, v/v) and the supernatants from the two extractions (400 μl for twice) were combined. The combined supernatants (100 μl) and 10 μl of internal standards (0.05 mg/ml of ^13^C_3_-^15^N-L-alanine, ^13^C_5_-^15^N-L-valine, ^13^C_6_-^15^N-L-leucine, and ^13^C_6_-^15^N-L-isoleucine) were dried under nitrogen, and the residues were first incubated with 20 mg/ml methoxyamine hydrochloride (30 μl) in pyridine at 37°C for 90 min and second derivatized with BSTFA with 1% TMCS (30 μl) at 70°C for 60 min prior to GC-MS metabolomics analysis.

Metabolomics instrumental analysis was performed using an Agilent 7890A gas chromatography system coupled to an Agilent 5975C inert MSD system (Agilent Technologies Inc., Santa Clara, CA, United States). An HP-5ms fused-silica capillary column (30 m × 0.25 mm × 0.25 μm; Agilent J&W Scientific, Folsom, CA, United States) was utilized to separate the derivatives. Detailed separation conditions and data collection were as described by Luo et al. (2019).

#### Sample Processing and LC-MS Analysis

Metabolite extraction was similar to GC-MS analysis, instead of the addition of 500 ng/ml lidocaine into the first extraction solution as internal standard. A mixture of 90 μl of combined supernatants and 10 μl of 0.1 mg/ml naproxen (internal standard) was injected at a volume of 6 μl for LC-MS analysis. The QC sample was prepared as a pool of extractions from all samples and applied throughout the experimental analysis.

Chromatographic separation was performed on an Agilent 1290 UHPLC system with a Waters UPLC HSS T3 column (2.1 mm × 100 mm, 1.8 μm) at a flow rate of 0.3 ml min^–1^ with a column temperature of 40°C. The mobile phases consisted of water (phase A) and acetonitrile (phase B), both with 0.1% formic acid (v/v). The linear gradient elution program was as follows: 0 min, 1% B; 1.5 min, 1% B; 13 min, 99% B; 16.5 min, 99% B; 16.6 min, 1% B; and hold to 20 min.

The eluents were analyzed in both electrospray positive (ESI+) and negative (ESI−) modes on a hybrid quadrupole time-of-light mass spectrometer (TripleTOF 6600 system, AB Sciex, Comcord, ON, Canada) equipped with a DuoSpray ion source (GS1 = 60 psi, GS2 = 60 psi, CUR = 30 psi, ion source temperature 550°C). The ion spray voltage floating (ISVF) was set to 5000 or −4000 V for ESI+ or ESI−, respectively. The declustering potential (DP) values were 60 and −60 V for ESI+ and ESI−, respectively. The TOF MS scan and data collection was performed as reported by Luo et al. (2019).

#### Data Preprocessing and Statistical Analysis

Peak picking, alignment, deconvolution, and further processing of raw GC-MS data were done with modifications from previously published protocols (Gao et al., 2010). The raw data of UPLC-QTOF-MS were transformed to mzXML format by ProteoWizard and then processed by XCMS and CAMERA packages in the R software platform. In XCMS, the peak picking [method = centWave, ppm = 15, peakwidth = c (5, 20, snthresh = 10)], alignment (bw = 6 and 3 for the first and second grouping, respectively), and retention time correction (method = loess) settings were used. In CAMERA, the annotations of isotope peak, adducts, and fragments were performed with default parameters.

The normalized data, including observations (sample name), variables (rt_mz), and normalized peak abundances, were analyzed with SIMCA software (version 13.0, Umetrics AB, Umeå, Sweden), where multivariate statistical analysis, such as principal component analysis (PCA) and partial least squares discriminant analysis (PLS-DA), were performed. The quality of models was described by the R^2^X (PCA) or R^2^Y and Q^2^ values (PLS-DA). To avoid model over-fitting, a default seven-round cross-validation was performed throughout to determine the optimal number of principal components. The values of R^2^X, R^2^Y, and Q^2^ were also used as indices to assess the robustness of a pattern recognition model (Luo et al., 2019).

Metabolites showing significant concentration differences between groups were identified using the combination of a variable importance in the projection (VIP) value > 1 from the orthogonal PLS-DA (OPLS-DA) model and a *p* < 0.05 from a univariate statistical analysis with Welch’s *t*-test (parametric test) on the metabolite’s data of normal distribution or Wilcoxon Mann–Whitney test (non-parametric test) on that of abnormal distribution. Fold-change was calculated as the binary logarithm of the average normalized peak intensity ratio between the two groups, where a positive value indicated that the average mass response of one specific group was upregulated compared to that of the other group (Luo et al., 2019).

#### Structural Identification of Differential Metabolites

For GC-MS data, the AMDIS software was applied to deconvolute mass spectra from raw GC-MS data, and the purified mass spectra were automatically matched against an in-house standard library that included retention time and mass spectra from the Golm Metabolome Database and Agilent Fiehn GC-MS Metabolomics RTL Library. For LC-MS data, the accurate *m/z* ratios of a precursor and its product ions were matched against databases including Metlin, Massbank, ReSpect, LipidBlast, and an in-house standard library. The threshold for matching similarity was 80%. In addition, previous publications were referenced in the structural identification of secondary metabolites such as phenolamides and benzoxazinoids (Dong et al., 2015; Bruijn et al., 2016). Metabolic pathway enrichment was analyzed using MetaboAnalyst 4.0.

### Production of Symptoms *in vitro*

*In vitro* production of symptom of black point was carried out according to the method reported by Williamson (1997), with some changes. Forty mature kernels from each of the four genotypes were individually imbibed in water for 10 min in 10 ml centrifuge tube. Then 30 imbibed kernels were placed in another 10 ml centrifuge tube containing 3 ml of 0.6% catechol (Sigma) for 10 min at 25°C, while the other 10 imbibed kernels placed in 0.3% H_2_O_2_ solution for 36 h. After that, 20 kernels soaked in catechol were further incubated in 0.3% H_2_O_2_ solution and 10 kernels in deionized water for 36 h. Kernels were observed visually at 10 h intervals.

## Results

### Symptoms of BP and BPF Kernels Inoculated With *B. sorokiniana*

**FIGURE 1:**
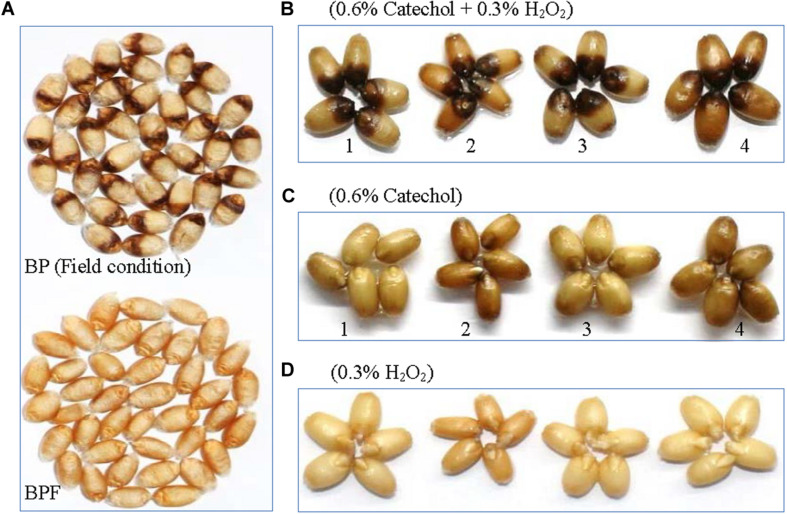
Kernels of PZSCL6 inoculated with *B. sorokiniana* under controlled field condition **(A)** and *in vitro* production of symptoms **(B–D)**. BP, black point- affected kernels; BPF, black point-free kernels. 1, 2, 3, and 4 represent wheat genotype Shannong4143 (resistant), SP1777-1-2 (resistant), Aifeng66 (susceptible), and PZSCL6 (susceptible), respectively.

Black point incidence of wheat genotype “PZSCL6” was more than 50% when inoculated with *B. sorokiniana* (Li et al., 2020). The symptoms on kernels of BP and BPF samples used for metabolomics and spectroscopic properties analysis are shown in Figure 1A. Discoloration characterized by a brown to black color at the embryo end of the kernel was visible with naked eye on BP kernels, which is the typical symptom of black point disease (Conner, 1989; Conner et al., 1992; Fernandez et al., 1994; Williamson, 1997; Mak et al., 2006; Toklu et al., 2008). In contrast, no discoloration was seen on BPF kernels selected.

### UV–Vis Spectra Analysis

**FIGURE 2:**
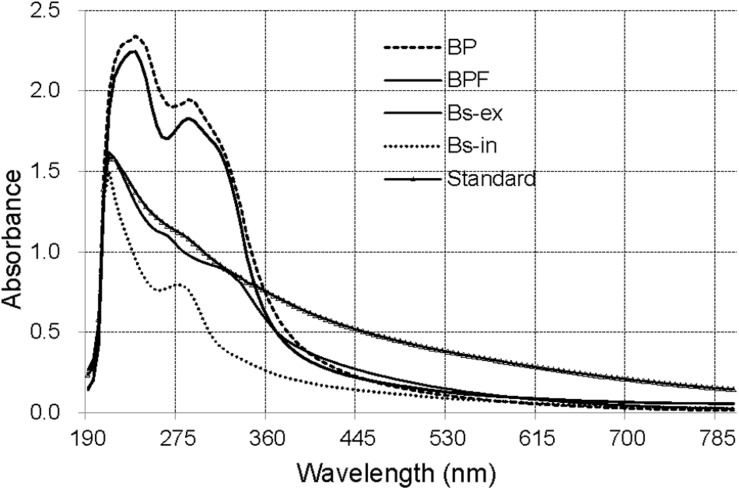
The UV–Vis spectra of extract from black point-affected (BP) and black point-free (BPF) kernels, extracellular and intracellular melanin from *B. sorokiniana* (BS-ex, BS-in), and a melanin standard.

The ultraviolet–visible (UV–Vis) absorbance (190–800 nm) spectra for solutions of extracts from BP and BPF kernels, extracellular and intracellular melanin from *B. sorokiniana* (Bs-ex, Bs-in), and a melanin standard are shown in Figure 2.

The absorbance of all the samples was strong over a wide spectral range and decreased with increasing wavelength. The synthetic melanin standard exhibited an expected peak at 215 nm (Wu et al., 2008). The maximal absorption peaks of Bs-ex and Bs-in were at 210 nm, similar to that of the melanin standard. UV–Vis absorbance spectra for extracts from wheat kernels were clearly different from those for the standard, and Bs-ex and Bs-in samples. UV–Vis absorbance spectra for BP and BPF were very similar, with a peak absorbance at 235 nm and a small shoulder at 280–300 nm. These results suggest that the blackening substance from BP kernels differs from synthetic melanin and the extracellular and intracellular melanin of *B. sorokiniana*.

### FT-IR Spectroscopy Analysis

**FIGURE 3:**
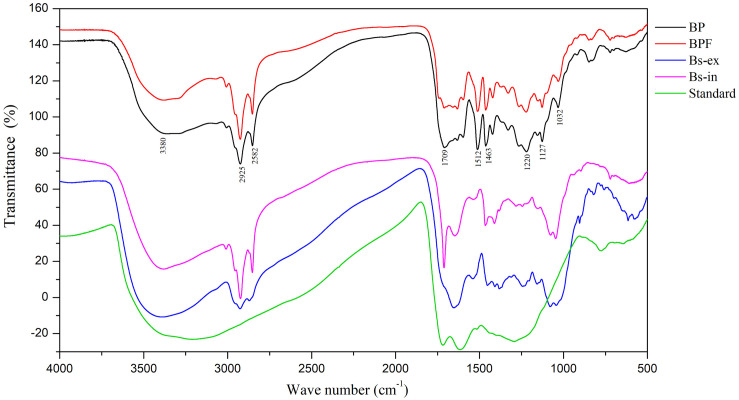
Fourier-transform infrared spectra of extracts from black point-affected (BP) and black point-free (BPF) kernels, extracellular and intracellular melanin from *B. sorokiniana* (BS-ex, BS-in), and a melanin standard.

Fourier-transform infrared spectroscopy is a useful tool for component studies of functional groups attached to polymer chains (Sava et al., 2001). The FT-IR spectrum of the melanin standard showed absorption peaks previously reported (Bilinska, 1996; Sava et al., 2001; Wu et al., 2008). A broad band at 3400–3300 cm^–1^ is indicative of stretching vibrations for –OH and –NH_2_ groups, and a strong absorption at 1650–1600 cm^–1^ corresponds to vibrations of aromatic groups (C = O or C = C, Figure 3).

Fourier-transform infrared results for the BP sample clearly differed from the melanin standard. Compared to the melanin standard, strong absorption peaks at 1620 cm^–1^ were absent and absorption peaks at 1220, 1463, 1512, and 2924 cm^–1^ were present. FT-IR spectra of the BP and BPF extracts were very similar (Figure 3). The broad, stretching peak at 3400–3300 cm^–1^ is indicative of –OH and –NH_2_ groups (Bilinska, 1996). The strong absorption peak at 2925 cm^–1^ and the absorption peak at 2852 cm^–1^ can be attributed to the stretching vibrations of carbon hydrogen bonds (C–H) or aliphatic C = H (Sava et al., 2001). The strong absorption peaks at 1512 and 1463 cm^–1^ can be attributed to vibrations of aromatic rings. A band at 1709 cm^–1^ indicates the presence of carbonyl groups. Generally, the absorption peak at 1220 cm^–1^ can be attributed to stretching of the C–O bond in phenol. The absorption around 600–860 cm^–1^ can be attributed to C-H out-plane vibrations in a benzene ring, and the weak absorption indicated that the benzene rings were substituted (Gonalves et al., 2012; Yao et al., 2012). These results identified alkyl, phenolic hydroxyl, and carboxyl functional groups in the extract of black point diseased kernels, indicative of a phenolic substance that is similar to the pepper seed melanin (Wang, 2014).

Compared to the Bs-ex sample, the BP sample includes two extra absorption peaks at 1463 and 1512 cm^–1^, but the absorption peak at 1651 cm^–1^ was absent. The FT-IR spectrum for Bs-in was similar to the sample from diseased kernels, but Bs-in absorption peaks at 1220 and 1512 cm^–1^ were absent in BP kernel sample. FT-IR spectroscopy showed that the bonding characteristics of extract from BP were similar to those from BPF, but were different from standard melanin and the intracellular and extracellular melanin from *B. sorokiniana*. These results suggest that the blackening substance was predominantly produced by wheat kernels, rather than by *B. sorokiniana*.

### Metabolites With Differential Content in BP and BPF Kernels

**FIGURE 4:**
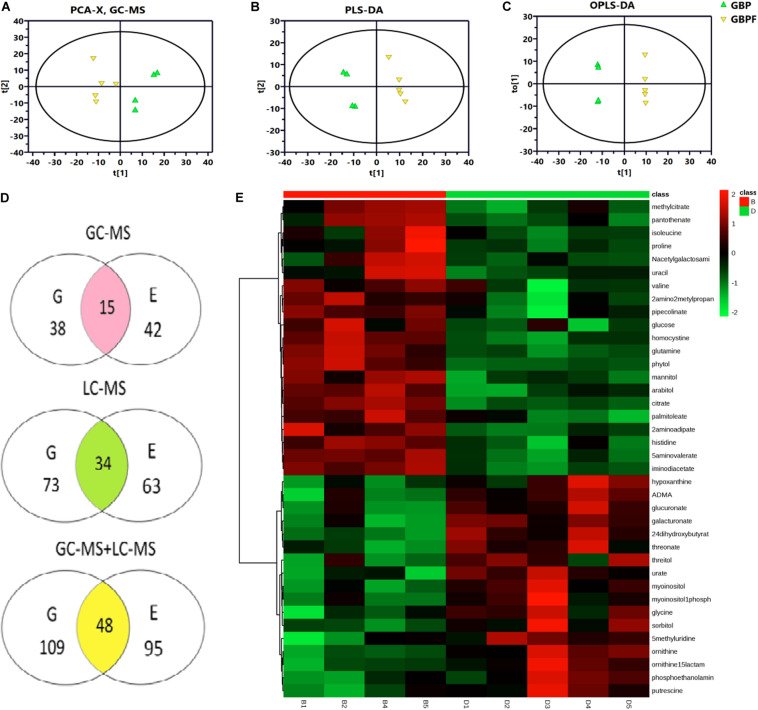
Metabolomics analysis of germ (G) and endosperm-bran (E) fractions between black point-affected (BP) and black point-free (BPF) wheat kernels. **(A–C)** Model scores plots comparing GC-MS data for EBP and EBPF kernels. **(A)** Principal component analysis (PCA; R^2^X = 0.565); **(B)** Partial least-squares-discriminate analysis (PLS-DA; R^2^Y = 0.998, Q^2^ = 0.930). **(C)** Orthogonal partial least-squares discriminant analysis (OPLS-DA, R^2^Y = 1.000, Q^2^ = 0.928). **(D)** Metabolites with differential content between BP and BPF identified by GC-MS and LC-MS. Values indicate the number of metabolites in a group. Data in the shaded cross-section count metabolites found in both G and E fractions. **(E)** Heat map of differential metabolites from the G fraction of BP and BPF as measured by GC-MS (*n* = 5). Each line represents a metabolite, and each column id represents a sample. B1–B5 and D1–D5 label BP and BPF replicates, respectively. Upregulated metabolites are shown in red, and downregulated metabolites are shown in green.

From PCA and PLS-DA results of LC-MS (electrospray negative, ESI−) for the four groups (GBP, EBP, GBPF, and EBPF), 20 samples (4 groups × 5 biological replicates) could be separated into four distinct clusters. PCA and PLS-DA results of electrospray positive (ESI+) and MC-MS were similar to those of LC-MS (ESI−) (Supplementary Figure 1). Then comparative analysis of EBP with EBPF and GBP with GBPF was performed. PCA (R^2^X = 0. 675), PLS-DA (R^2^Y = 0.997, Q^2^ = 0.784), and OPLS-DA (R^2^Y = 1.000, Q^2^ = 0.850) of GBP and GBPF showed the identified differences between samples of GC-MS data set. The PCA, PLS-DA, and OPLS-DA of EBP and EBPF for GC-MS and LC-MS are shown that R^2^X in PCA ranged from 0.565 to 0.727, and R^2^Y and Q^2^ in PLS-DA and OPLS-DA ranged from 0.980 to 1.000 and 0.782 to 0.978, respectively (Figures 4A–C and Supplementary Table 1). Model scores plots showed that different samples were approximately clustered on the basis of black point symptoms, suggesting that the model could clearly and reproducibly classify BP and BPF groups.

Variable importance in the projection threshold from OPLS-DA models (>1) were combined with univariate statistical analysis (*p* < 0.05) to asses GC-MS data and identified 38 and 42 metabolites with differential content in germ and endosperm-bran fractions, respectively (Figure 4D and Supplementary Tables 2, 3). Through LC-MS analysis, 73 and 63 metabolites with differential content were found in germ and endosperm-bran fractions, respectively (Supplementary Tables 4, 5). Heat map analysis was used to depict the levels of differential metabolites in every sample (Figure 4E and Supplementary Figures 2–4).

Combining GC-MS and LC-MS methods, 109 and 95 metabolites with differential content were found in germ fraction and endosperm-bran fraction, respectively (Supplementary Tables 6, 7). A total of 156 metabolites with differential content between BP and BFP kernel were found, including 48 metabolites with differential content in both the germ and the endosperm-bran fractions (Supplementary Table 8). Thirty-eight metabolites with content that was twofold higher or lower in BP than that in BPF kernels are shown in Table 1. Of these 156 metabolites, 70 metabolites were detected with significantly lower content and 69 metabolites with significantly higher content in BP kernels compared to BPF kernels. Other 17 metabolites showed inconsistent content changes in germ and endosperm-bran fractions.

**TABLE 1:** Thirty-eight metabolites with content that was twofold higher or lower in black point-affected (BP) kernels than that in black point-free (BPF) kernels.

Metabolites*^*a*^*	VIP*^*b*^*	*p*-value^*c*^	FC*^*d*^*	BP/BPF*^*e*^*	Sample *^*f*^*	Method*^*f*^*
2,4-Dihydroxybutyric acid	1.38–1.72	0.0218–0.0014	−1.11 to 1.41	0.46–0.38	E + G	GC-MS
Arabitol	1.62	0.0029	1.05	2.07	E	GC-MS
Aspartic acid	1.72–1.54	0.0007–0.0049	−1.17 to 1.18	0.44–0.44	E	GC-MS/LC-MS
Citric acid	1.89–4.44	0.0000–0.0206	1.05–0.61	2.07–1.53	E + G	GC-MS/LC-MS
DG 36:4; DG(18:2/18:2/0:0)	5.53	0.0297	−1.76	0.30	G	LC-MS
DIMBOA-hex-hex	20.98	0.0024	1.71	3.27	G	LC-MS
FA(C18:3) peak3	9.14	0.0108	1.61	3.05	G	LC-MS
FA(C18:4)	12.57	0.0425	1.10	2.14	G	LC-MS
Fumaric acid	1.66–5.30	0.0017–0.0035	−1.19 to 0.99	0.44–0.50	E	GC-MS/LC-MS)
Glucaric acid	1.26	0.0441	−1.30	0.41	E	GC-MS
Gluconic acid	1.6	0.0037	1.26	2.39	E	GC-MS
Glutamine	1.59–4.09	0.0001–0.0117	0.96–1.64	1.94–3.12	E + G	GC-MS/LC-MS)
Guanosine	1.34	0.0281	1.12	2.17	E	GC-MS
HDMBOA-hex-hex	2.1	0.0198	3.74	13.36	G	LC-MS
Histamine	1.19	0.0208	1.65	3.14	G	LC-MS
Iditol	5.25–6.87	0.0076–0.0393	0.76–1.02	1.69–2.03	E + G	LC-MS
Linoleoyl Ethanolamide (LEA)	5.43	0.0078	−1.09	0.47	E	LC-MS
Malic acid	1.66–0.71	0.0017–0.0029	−1.02 to 0.95	0.49–0.52	E	GC-MS/LC-MS)
Methylsuccinic acid	1.47	0.0115	−1.08	0.47	E	GC-MS
MGDG 36:6; MGDG(18:3/18:3)	4.82	0.0009	−1.19	0.44	G	LC-MS
N1,N10-Caffeoylferuloylspermidine	2.46–4.89	0.0011–0.0121	1.57–2.96	2.97–7.78	E + G	LC-MS
N1,N10-Dicaffeoylspermidine	1.34–4.28	0.0108–0.0059	1.54–2.27	2.91–4.82	E + G	LC-MS
N1,N10-Diferuloylspermidine	4.83–12.61	0.0002–0.0175	1.36–2.84	2.57–7.16	E + G	LC-MS
N1,N10-Di-p-coumaroylspermidine	2.02–5.07	0.0001–0.0116	2.72–2.78	6.59–6.87	E + G	LC-MS
N1,N10-p-Coumaroylferuloylspermidine	9.45–9.54	0.0000–0.0093	1.80–2.83	3.48–7.11	E + G	LC-MS
N-Feruloylspermidine	6.09	0.0000	2.15	4.44	G	LC-MS
N-p-Coumaroyl-2-hydroxyputrescine	3.41–5.67	0.0021–0.0333	2.31–3.48	4.96–11.16	E + G	LC-MS
N-p-Coumaroylputrescine	1.11–1.21	0.0012–0.0323	0.99–1.07	1.99–2.10	E + G	LC-MS
N-p-Coumaroylspermidine	1.35–1.99	0.0058–0.0321	1.99–0.60	1.52–3.97	E + G	LC-MS
PC 34:2; PC(18:2/16:0)	4.78	0.0242	1.45	2.73	G	LC-MS
p-Coumaric acid	1.26–5.06	0.0140–0.1450	0.65–1.68	1.57–3.20	E + G	GC-MS/LC-MS)
Phytol	1.85	0.0001	1.53	2.89	G	GC-MS
PI 36:6; PI(18:3/18:3)	1.26–2.68	0.0024–0.0058	1.05–1.45	2.07–2.73	E + G	LC-MS
Proline	1.29–1.43	0.0235-0.0366	1.08–1.15	2.11–2.22	E + G	GC-MS
Quinic acid	1.76–1.49	0.0003–0.0016	−2.08 to 1.33	0.24–0.40	E	GC-MS/LC-MS)
Succinic acid	2.10–1.77	0.0002–0.0057	−1.18 to 1.17	0.44–0.44	E	GC-MS/LC-MS)
Tryptophan	1.24–14.09	0.0487–0.0496	−1.76 to 1.37	0.29–0.30	E	GC-MS/LC-MS)
Uric acid	1.56	0.0051	−2.33	0.20	E	GC-MS

**^*a*^*DG, diacyl glycerol; DGDG, digalactosyl diacylglycerol; FA, fatty acid; GlcCer, glucosylceramide; MGDG, monogalactosyl diacylglycerol; PC, phosphatidylcholine; PI, phosphatidylinositol. *^*b*^*VIP, variable importance in the projection, was obtained from the OPLS-DA model. *^*c*^*The *p*-value was calculated from univariate statistical analysis. *^*d*^*FC (fold change) was calculated as a binary logarithm of the average mass response (normalized peak area) ratio between BP vs BPF, where a positive and negative value means that the average mass response of the metabolite in BP is larger and lower than that in BPF, respectively. *^*e*^*BP/BPF, the normalized peak area ratio between BP and BPF groups. *^*f*^*E, endosperm-bran fraction, G, germ fraction; GC-MS, gas chromatography-mass spectrometer; LC-MS, liquid chromatograph-mass spectrometer.*

**FIGURE 5:**
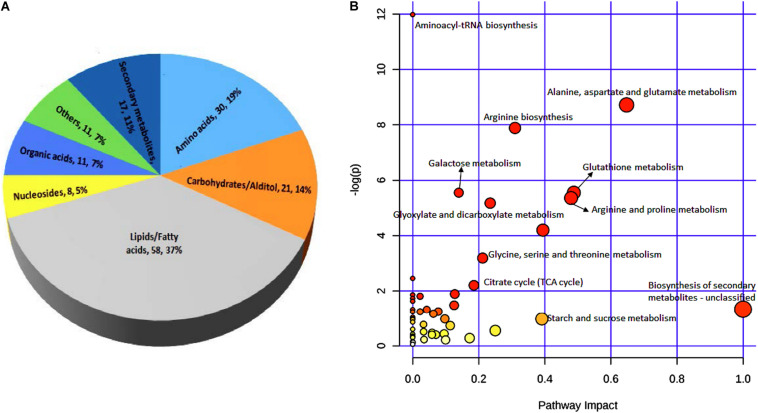
Classification of 156 metabolites **(A)** and KEGG pathway analysis **(B)** of differential metabolites between black point-affected and black point-free wheat kernels. In **B**, larger circles indicate greater pathway enrichment, and darker colors indicate larger pathway impact values.

These 156 differential metabolites were divided into seven major groups based on their chemical characteristics. The largest group contained 58 metabolites (37% of all metabolites detected) that are lipids and fatty acids. The second largest group contained 30 metabolites (19%) that are amino acids. The other five groups contained 21 metabolites (14%), 17 metabolites (11%), 11 metabolites (7%), 11 metabolites (7%), and eight metabolites (5%) that are characterized as carbohydrates/altiol, secondary metabolites, organic acids, other chemicals, and nucleotides, respectively (Figure 5A).

In both germ and endosperm-bran fractions, the amounts of ferulic acid and p-coumaric acid in BP sample were significantly greater than those in BPF sample. In addition, 11 metabolites with significantly higher content in BP kernels, including N1, N10-Caffeoylferuloylspermidine, N1, N10-Diferuloylspermidine, and N-p-Coumaroylputrescine, were phenolamide compounds; and four metabolites with significantly higher content in BP kernels were benzoxazinone derivatives (Supplementary Table 8). To develop a better understanding of the underlying metabolome changes occurred in BP kernels, significant metabolites were mapped onto metabolic pathways. KEGG pathway analysis of differential metabolites showed significant enrichment of metabolites involved in starch and sucrose, amino acid, glutathione, glyoxylate, dicarboxylate metabolism, and biosynthesis of secondary metabolites (Figure 5B).

### Symptoms of Black Point Produced *in vitro*

After 10 min incubation in the phenolic substrate (0.6% catechol), followed by 36 h incubation in 0.3% H_2_O_2_, all mature wheat kernels of four genotypes (both susceptible and resistant) showed blackening symptoms of black point covering approximately one-third of the grain at the germ, with a clear demarcation between the blacken and the symptomless portion of the kernels (Figure 1B). No discoloration symptoms appeared on any of the kernel incubated in catechol or H_2_O_2_ alone (Figures 1C,D), and the symptoms did not occur even if the incubation time was extended to 60 h. Kernels soaked in catechol alone showed slight browning with clear distinction to black point symptoms. There is no difference in the intensity or size of the discoloration between resistant and susceptible genotypes.

## Discussion

### Blackening Substance of Black Point Was Produced in Wheat Kernels

It remains to be determined whether black point is primarily pathogen-induced (Southwell et al., 1980; Rees et al., 1984; Conner et al., 1996; Kumar et al., 2002) or induced by environmental conditions (Conner and Davidson, 1988; Conner, 1989; Conner et al., 1992; Fernandez et al., 1994; Kumar et al., 2002; Moschini et al., 2006; Walker, 2011). Our previous study indicated that pathogens could significantly increase the incidence of black point (Xu et al., 2018), and the incidence was also affected by environmental variations between different years (Li et al., 2019a,b). It seems that the pathogen causes black point, and the appropriate environmental conditions will enhance the severity of this disease.

Beyond identifying the cause of black point, understanding how the discoloration occurs is an outstanding problem of interest. Some researchers proposed that discoloration is the result of fungal colonization of kernels, including *A. alternata*, *B. sorokiniana*, *Exserohilum rostratum*, and *F. proliferatum* (Southwell et al., 1980; Rees et al., 1984; Conner et al., 1996; Kumar et al., 2002). Other researchers reported that blackening is probably the result of biochemical changes within the kernel (Williamson, 1997; Walker and Ferrar, 1998; Walker, 2011). In this study, the spectroscopic properties of blackening substance extracted from the diseased area of BP kernels were discovered markedly different from those of synthetic melanin and melanin collected from the pathogen *B. sorokiniana*. Furthermore, the spectroscopic properties of the extract from BP kernels were very similar to those made from an extract of the analogous part of BPF kernels. These results indicate that the blackening substance of black point comes from the wheat kernel instead of pathogenic fungi (*B. sorokiniana*). This blackening substance was also synthesized in BPF kernels with a lower amount than in BP kernels (data not shown). It suggested that the formation of blackening substance causing black point should be a natural reaction to the fungal infection. In the kernels inoculated with *B. sorokiniana*, more blackening substance was accumulated, which resulted in the visible symptoms of black point.

### Enzymatic Browning Bring About the Production of Blackening Substance

In this study, results of FT-IR spectroscopy identified alkyl, phenolic hydroxyl, and carboxyl functional groups in the extract from BP kernels, indicative of a phenolic substance. Metabolomics analysis further showed that significantly higher content of ferulic and p-coumaric acid in BP kernels, which indicated that enough phenolic substrate for enzymatic browning is helpful to produced more black pigments than that in BPF kernels. In addition, symptoms of black point could be produced *in vitro* when asymptomatic wheat kernels were treated with phenol substrate (catechol) and H_2_O_2_ solution, which was consistent with the results of Williamson (1997). These results indicated that the enzymatic browning in wheat kernels brings about the production of blackening substance causing black point. The discoloration did not appear when the kernels were incubated in the phenol substrates and H_2_O_2_ alone even if treatment time is enough, which were also reported by Williamson (1997). The requirement for H_2_O_2_ suggests that POD were involved in the discoloration reaction of black point. The requirement for supplement of exogenous phenol substrate suggests the insufficient phenol content in BPF kernels limited the production of blackening substance causing black point.

Enzymatic browning is a characteristic reaction in plant tissues under stressful or harmful condition. It involves two complex processes. First, the phenolic substrates is oxidized under the catalysis of polyphenol oxidase (PPO, EC 1.10.3.1) and POD, and then the oxidized products are transformed to black pigments, like melanins and quinnes (Walker and Ferrar, 1998; Tomás-Barberán and Espin, 2001). POD enzymes catalyze the oxidation of various substrates, including ferulic and p-coumaric acid (the main phenolic acids found in monocotyledonous crops), into dark-colored products (Sosulski et al., 1982; Rasmussen et al., 1997). Phenolic acids are natural compounds in wheat kernels (Kim et al., 2006). In addition to being substrates for POD and PPO in enzymatic browning process, phenolic acids have been found to be strong antioxidants (Kim et al., 2006) and act as germination inhibitors (Weidner et al., 1999).

### The Reason for Discoloration of Black Point Was Associated With Enzymatic Browning

Certain environmental stresses during grain filling, such as infection by pathogen, wheat pre-germination, or disruption of the immature caryopsis, might cause POD and PPO release and catalyze the oxidation of phenols to form quinones, which are highly reactive and produce insoluble polymers associated with discoloration symptoms (Barz and Koster, 1981; Cochrane, 1994). In response to stress encountered during kernel development, there is activation of a complex defense mechanism in plants that includes a scavenging system to cope with excessive reactive oxygen species (ROS) and the accumulation of phenylpropanoid compounds that directly act as defense factors or may act as precursors for the synthesis of lignin, suberin, and other wound-induced polyphenolic barriers (Dixon and Paiva, 2017).

Mak et al. (2006) found lower levels of “stress” class proteins (products of genes associated with stress, disease, and defense) in BP kernels through proteomic analysis, indicting weaker ability in response to stress in BP kernels. Owing to relatively smaller amounts of “stress” proteins, BP kernels need a lot of phenolic compounds and enzymes or activate other mechanisms to cope with stresses and synthesize metabolites with antioxidant functions. In this study, metabolomics analysis showed significantly higher content of ferulic and p-coumaric acid in BP kernels, which could be used against ROS that results from *B. sorokiniana* infection and provided enough phenolic substrate for enzymatic browning. In addition, metabolomics analysis also showed that the content of 11 phenolamide compounds and four benzoxazinone derivatives were significantly higher in BP kernels than in BPF kernels. Phenolamides, a major group of secondary metabolites resulting from the conjugation of a phenolic moiety with polyamines, have been reported throughout the plant kingdom and are considered to be either products of polyamine catabolism or phenolic storage forms (Bassard et al., 2010) with specific functions in plant development and defense. In addition, phenolamides have also been described as bioactive compounds with antiviral, antibacterial, antifungal, and insecticidal activities (Walters et al., 2002; Bassard et al., 2010). Benzoxazinoids are nitrogen-containing secondary metabolites found in poaceous plants, including wheat and maize (Niemeyer, 2009). Among the three groups of benzoxazinoids (benzoxazolinones: BOA and MBOA; lactams: HBOA, HMBOA, and their glycosides; hydroxamic acids: DIBOA, DIMBOA, and their glycosides), DIMBOA and its glycosides are predominant in maize and wheat (Jonczyk et al., 2008; Tanwir et al., 2017). Benzoxazinoids are stored in cells as glucosides. When the cytoarchitecture is ruptured, the active aglycons, DIBOA, and/or DIMBOA are released enzymatically and act as important factors in host-plant resistance to microbial disease and insects (Niemeyer, 1988, 2009; Sicker et al., 2000). These results indicated that due to the insufficient ability to deal with ROS caused by infection of *B. sorokiniana* in BP kernels, the phenolic compounds and activities of enzymes related to enzymatic browning were increased, subsequently blacking substances causing symptom of black point were accumulated. Compared to the resistant wheat cultivar, the activities of POD and PPO were significantly increased after inoculated by *B. sorokiniana* in the cultivar susceptible to black point (unpublished data).

Based on the results of this study, the biochemical pathways, including phenylpropanoid metabolism, oxidation of phenolic acids, to increase phenols and quinones associated with enzymatic browning, may accelerate the development of black point symptoms. Although POD, PPO, H_2_O_2_, and the phenols, which were considered necessary for enzymatic browning, could be found in wheat kernels (Cochrane, 1994), the mechanism by which they could combine to create discoloration remains to be fully understood. Identifying the specific phenol oxidase that accelerates black point symptoms and understanding how stress conditions or cellular disruption caused by specific environmental conditions release the peroxidases will be necessary to further understand the molecular mechanism of browning. Future research will concentrate on these issues in order to understand what is happening within kernels during black point development under the condition of inoculation of *B. sorokiniana*.

## Conclusion

The spectroscopic properties of extracts from the blackening parts of BP kernels were similar to those from the analogous parts of BPF kernels but different from those of melanin from fungi (*B. sorokiniana*). With the method combines GC-MS and LC-MS, we identified 156 differential metabolites between BP and BPF kernels from a susceptible wheat genotype inoculated with *B. sorokiniana* under controlled field condition. Among the 156 metabolites, the content of phenolic acids in diseased kernels was significantly higher than that of asymptomatic kernels in both germ and endosperm-bran fractions. The symptom of black point could be produced *in vitro* with supplement of phenol substrate (catechol) and H_2_O_2_. This indicates that enzymatic browning in wheat kernels brings about the symptom of black point.

## Data Availability Statement

All datasets generated for this study are included in the article/Supplementary Material.

## Author Contributions

QL performed the experiment and wrote the manuscript. KX and SW completed the metabolomics and UV–Vis absorbance analysis. ML and YJ cultured the pathogen, completed the inoculation, and extracted blacken substance. XL did FT-IR analysis. JN analyzed the data. CW designed the experiment and edited the manuscript. All authors approved the manuscript.

## Conflict of Interest

Shanghai ProfLeader Biotech Co., Ltd. is acknowledged for assistance with the metabolomics experiments and data analysis. The authors declare that the research was conducted in the absence of any commercial or financial relationships that could be construed as a potential conflict of interest.

## Supplementary Material

Supplementary Figure 1 Principal component analysis (PCA) and partial least squares discriminant analysis (PLS-DA) for four group samples in LC-MS **(A–H)** and GC-MS **(I–L)**. **(A,B)** PCA of electrospray negative (ESI−), R^2^X = 0.786; **(C,D)** PLS-DA of ESI−, R^2^Y = 0.995, Q^2^ = 0.911; **(E,F)** PCA of electrospray positive (ESI+), R^2^X = 0.766; **(G,H)** PLS-DA of ESI + , R^2^Y = 0.987, Q^2^ = 0.886; **(I,J)** PCA of GC-MS, R^2^X = 0.674; **(K,L)** PLS-DA of GC-MS, R^2^Y = 0.988, Q^2^ = 0.882; *n* = 5.

Supplementary Figure 2 Heat map of differential metabolites from the endosperm-bran fraction of diseased and asymptomatic kernels as measured by GC-MS (*n* = 5). Each line means a metabolite, and each column id for each sample. A1-A5 represent replicates of diseased group and C1-C5 represent replicates of asymptomatic group. The upregulated metabolites are shown in red color, whereas the downregulated metabolites are presented in green color. GC-MS, gas chromatography-mass spectrometer.

Supplementary Figure 3 Heat map of differential metabolites from the endosperm-bran fraction of diseased and asymptomatic kernels as measured by LC-MS (*n* = 5). Each line means a metabolite, and each column id for each sample. A1–A5 represent replicates of diseased group and C1–C5 represent replicates of asymptomatic group. The upregulated metabolites are shown in red color, whereas the downregulated metabolites are presented in green color. LC-MS, liquid chromatograph-mass spectrometer.

Supplementary Figure 4 Heat map of differential metabolites from the germ fraction of diseased and asymptomatic kernels as measured by LC-MS (*n* = 5). Each line means a metabolite, and each column id for each sample. B1–B5 represent replicates of diseased group and D1–D5 represent replicates of asymptomatic group. The upregulated metabolites are shown in red color, whereas the downregulated metabolites are presented in green color. LC-MS, liquid chromatograph-mass spectrometer.

Supplementary Table 1 Principal component analysis (PCA), partial least squares discriminant analysis (PLS-DA), and orthogonal partial least-squares discriminant analysis (OPLS-DA) of the samples from diseased and asymptomatic kernels.

Supplementary Table 2 Thirty-eight metabolites identified by GC-MS in the germ fraction with differential content in black point-affected (BP) and black point-free (BPF) kernels.

Supplementary Table 3 Forty-two metabolites identified by GC-MS in the endosperm-bran fraction with differential content in black point-affected (BP) and black point-free (BPF) kernels.

Supplementary Table 4 Seventy-three metabolites identified by LC-MS in the germ fraction with differential content between black point-affected (BP) and black point-free (BPF) kernels.

Supplementary Table 5 Sixty-three metabolites identified by LC-MS in the endosperm-bran fraction with differential content between black point-affected (BP) and black point-free (BPF) kernels.

Supplementary Table 6 One hundred and nine metabolites identified by combining LC-MS and MC-MS in the germ fraction with differential content between black point-affected (BP) and black point-free (BPF) kernels.

Supplementary Table 7 Ninety-five metabolites identified by combining LC-MS and MC-MS in the endosperm-bran fraction with differential content between black point-affected (BP) and black point-free (BPF) kernels.

Supplementary Table 8 One hundred and fifty-six metabolites identified by combining LC-MS and MC-MS with differential content between black point-affected (BP) and black point-free (BPF) kernels.
