# Supplementary material for: Enzymatic Browning in Wheat Kernels Produces Symptom of Black Point Caused by Bipolaris sorokiniana
Source: Front Microbiol. 2020 Dec 9;11:526266. doi: 10.3389/fmicb.2020.526266 (PMC7756095; doi:10.3389/fmicb.2020.526266)
Supplement: Supplementary Figure 1 — Principal component analysis (PCA) and partial least squares discriminant analysis (PLS-DA) for four group samples in LC-MS (A–H) and GC-MS (I–L). (A,B) PCA of electrospray negative (ESI−), R2X = 0.786; (C,D) PLS-DA of ESI−, R2Y = 0.995, Q2 = 0.911; (E,F) PCA of electrospray positive (ESI+), R2X = 0.766; (G,H) PLS-DA of ESI + , R2Y = 0.987, Q2 = 0.886; (I,J) PCA of GC-MS, R2X = 0.674; (K,L) PLS-DA of GC-MS, R2Y = 0.988, Q2 = 0.882; n = 5. [file Data_Sheet_1.pdf]

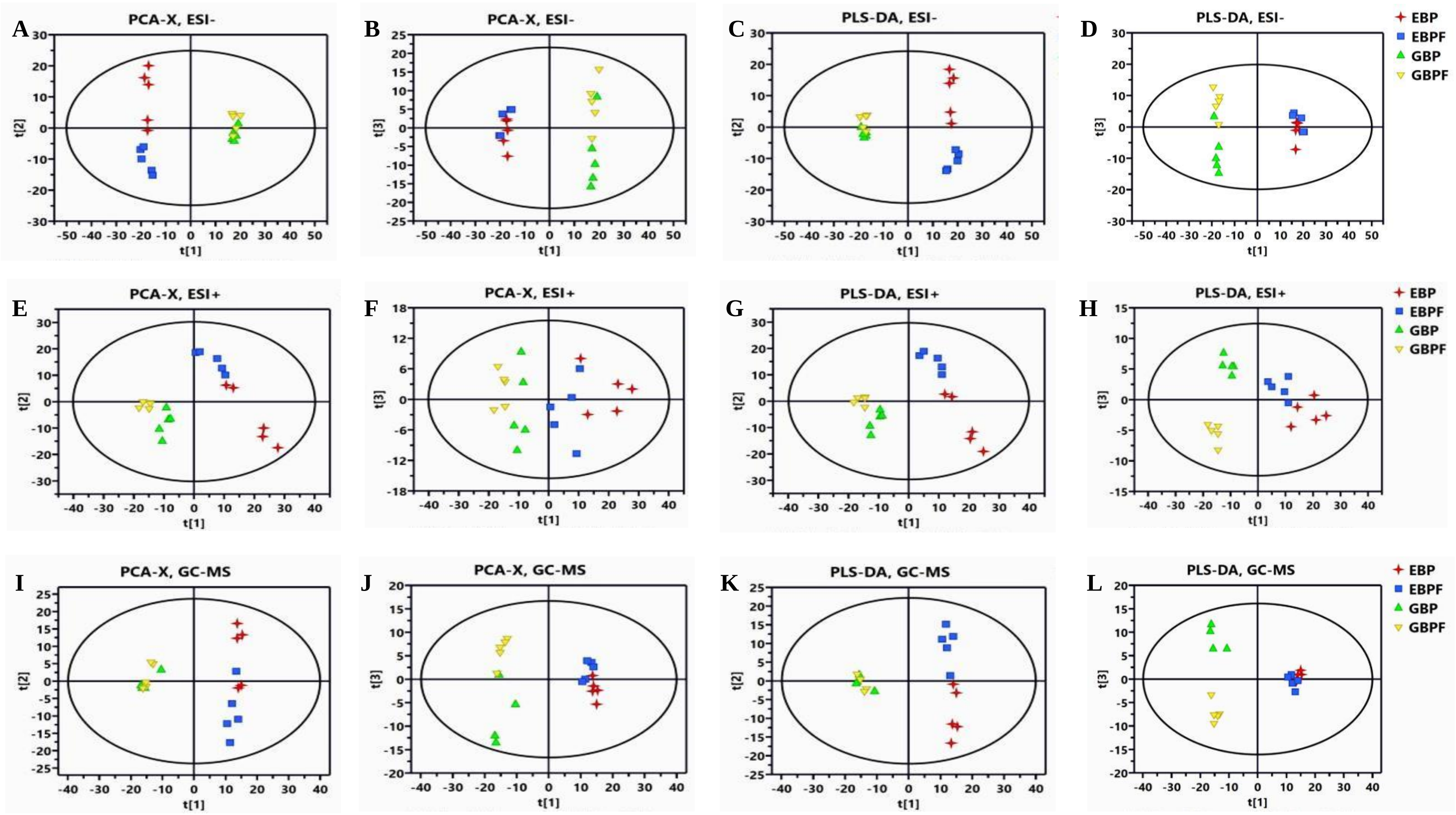

Supplementary Figure S1 Principal component analysis (PCA) and partial least squares discriminant analysis (PLS-DA) for four groups in LC-MS (A-H) and GC-MS (I-L).

A and B, PCA of electrospray negative (ESI-), R^2X =0.786; C and D, PLS-DA of ESI-, R^2Y = 0.995, Q^2 = 0.911; E and F, PCA of electrospray positive (ESI+), R^2X =0.766; G and H, PLS-DA of ESI+, R^2Y = 0.987, Q^2 = 0.886; I and J; PCA of GC-MS, R^2X =0.674; K and L, PLS-DA of GC-MS, R^2Y = 0.988, Q^2 = 0.882); n = 5.

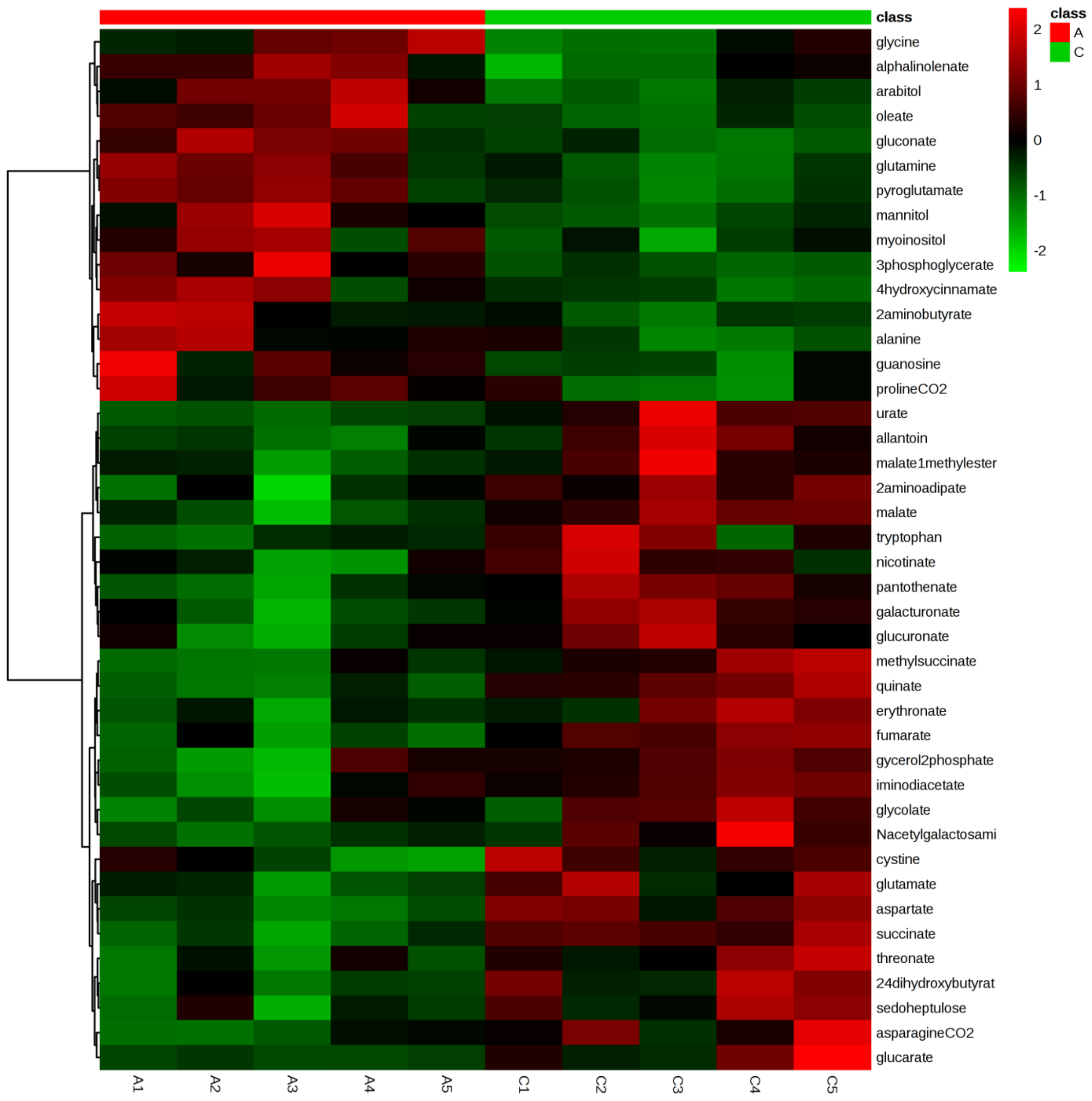

Supplementary Figure S2 Heat map of differential metabolites from the endosperm-bran fraction of diseased and asymptomatic kernels as measured by GC-MS (n = 5).

Each line means a metabolite, and each column id for each sample. A1−A5 represent replicates of diseased group and C1−C5 represent replicates of asymptomatic group. The up-regulated metabolites are shown in red color, whereas the down-regulated metabolites are presented in green color. GC-MS= Gas Chromatography-Mass Spectrometer.

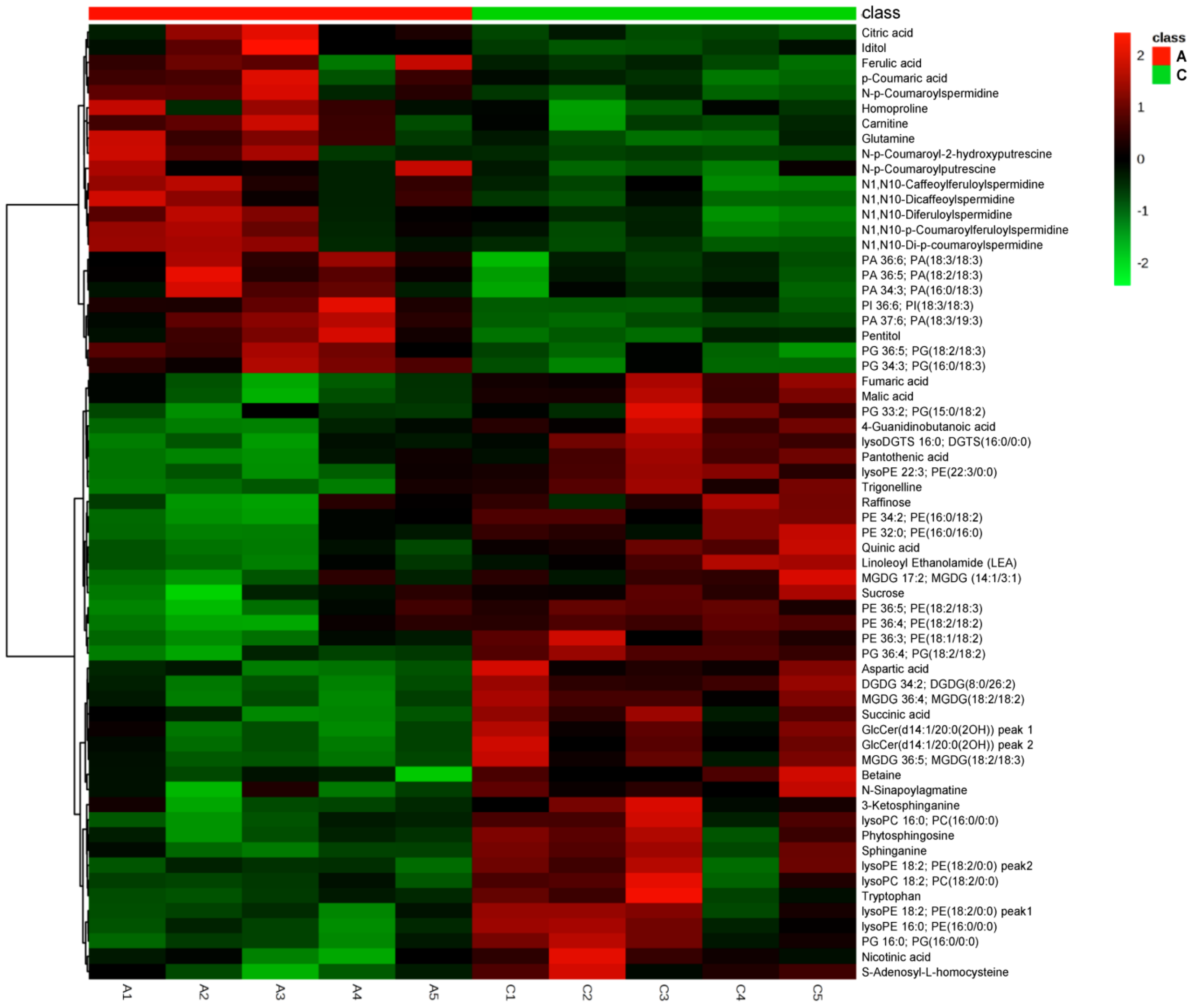

Supplementary Figure S3 Heat map of differential metabolites from the endosperm-bran fraction of diseased and asymptomatic kernels as measured by LC-MS (n = 5).

Each line means a metabolite, and each column id for each sample. A1−A5 represent replicates of diseased group and C1−C5 represent replicates of asymptomatic group. The up-regulated metabolites are shown in red color, whereas the down-regulated metabolites are presented in green color. LC-MS= Liquid Chromatograph-Mass Spectrometer.

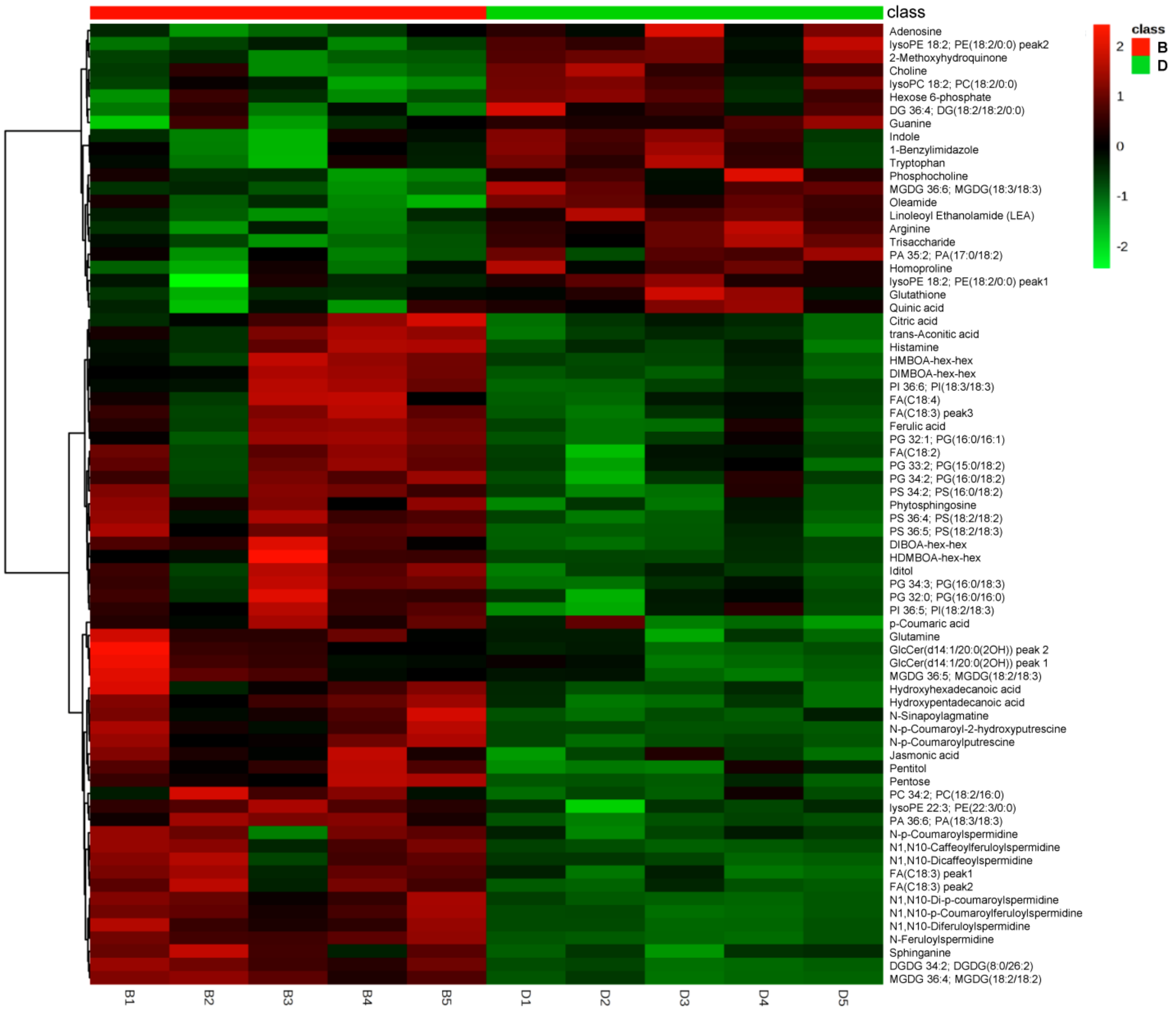

Supplementary Figure S4 Heat map of differential metabolites from the germ fraction of diseased and asymptomatic kernels as measured by LC-MS (n = 5).

Each line means a metabolite, and each column id for each sample. B1−B5 represent replicates of diseased group and D1−D5 represent replicates of asymptomatic group. The up-regulated metabolites are shown in red color, whereas the down-regulated metabolites are presented in green color. LC-MS= Liquid Chromatograph-Mass Spectrometer.
