# Supplementary material for: Enzymatic Browning in Wheat Kernels Produces Symptom of Black Point Caused by Bipolaris sorokiniana
Source: Front Microbiol. 2020 Dec 9;11:526266. doi: 10.3389/fmicb.2020.526266 (PMC7756095; doi:10.3389/fmicb.2020.526266)
Supplement: Supplementary Table 1 — Principal component analysis (PCA), partial least squares discriminant analysis (PLS-DA), and orthogonal partial least-squares discriminant analysis (OPLS-DA) of the samples from diseased and asymptomatic kernels. [file Data_Sheet_2.pdf]

Supplementary Table S1 Principal component analysis (PCA), partial least squares discriminant analysis (PLS-DA) and orthogonal partial least-squares discriminant analysis (OPLS-DA) of the samples from diseased and asymptomatic kernels

Group [a]	Analysis method [bc]	PCA	PLS-DA		OPLS-DA	
		R^2X	R^2Y	Q2	R^2Y	Q2
GBP/GBPF	GC-MS	0.675	0.997	0.784	1.000	0.850
	LC-MS (ESI+)	0.605	0.991	0.924	0.999	0.940
	LC-MS (ESI-)	0.673	0.989	0.792	0.999	0.782
EBP/EBPF	GC-MS	0.565	0.998	0.930	1.000	0.928
	LC-MS (ESI+)	0.727	0.980	0.901	0.997	0.934
	LC-MS (ESI-)	0.591	0.996	0.927	1.000	0.978

a EBP and GBP = endosperm-bran and germ fraction of black point-affected kernels, respectively. EBPF and GBPF = endosperm-bran and germ fraction of black point-free kernels, respectively.
b GC-MS= Gas Chromatography-Mass Spectrometer, LC-MS= Liquid Chromatograph-Mass Spectrometer.
c ESI+ = electrospray positive mode, ESI- = electrospray negative modes.

Supplementary Table S2 Thirty-eight metabolites identified by GC-MS in the germ fraction with differential content in black point-affected (BP) and black point-free (BPF) kernels

No.	Metabolites	VIP [a]	*p*-value [b]	FC [c]	HMDB	KEGG
1	Citric acid	1.89	0.0000	0.61	HMDB00094	C00158
2	Glucose	1.43	0.022	0.37	HMDB00122	C00031
3	Proline	1.43	0.024	1.15	HMDB00162	C00148
4	Putrescine	1.38	0.031	-0.82	HMDB01414	C00134
5	Ornithine	1.63	0.004	-0.6	HMDB00214	C00077
6	Glutamine	1.83	0.0001	1.18	HMDB00641	C00064
7	Threonic acid	1.55	0.009	-0.62	HMDB00943	C01620
8	Pantothenic acid	1.58	0.007	0.38	HMDB00210	C00864
9	Homocystine	1.82	0.000	0.5	HMDB00676	C01817
10	Palmitoleic acid	1.60	0.006	0.65	HMDB03229	C08362
11	Mannitol	1.75	0.001	0.59	HMDB00765	C00392
12	Sorbitol	1.41	0.025	-0.68	HMDB00247	C00794
13	Myo-inositol	1.55	0.009	-0.21	HMDB00211	C00137
14	Myo-inositol-1-phosphate	1.32	0.043	-0.71	HMDB00213	C04006
15	Phosphoethanolamine	1.29	0.049	-0.61	HMDB00224	C00346
16	Glycine	1.43	0.023	-0.4	HMDB00123	C00037
17	Histidine	1.71	0.001	0.7	HMDB00177	C00135
18	2-Aminoadipic acid	1.74	0.001	0.84	HMDB00510	C00956
19	Pipecolinic acid	1.53	0.011	0.53	HMDB00070	C00408
20	5-Aminovaleric acid	1.84	0.000	0.83	HMDB03355	C00431
21	Arabitol	1.75	0.001	0.48	HMDB00568	C01904
22	Glucuronic acid	1.50	0.014	-0.56	HMDB00127	C00191
23	Hypoxanthine	1.37	0.033	-0.32	HMDB00157	C00262
24	Uric acid	1.56	0.008	-0.72	HMDB00289	C00366
25	Uracil	1.39	0.029	0.55	HMDB00300	C00106
26	Isoleucine	1.32	0.043	0.46	HMDB00172	C00407
27	Valine	1.35	0.036	0.31	HMDB00883	C00183
28	2,4-Dihydroxybutyric acid	1.72	0.001	-1.41	HMDB00360	
29	2-Amino-2-metyl-propanoic acid	1.48	0.017	0.54		
30	5-Methyluridine	1.35	0.038	-0.41	HMDB00884	
31	ADMA(asymmetric dimethylarginine)	1.52	0.012	-0.42	HMDB01539	C03626
32	Galacturonic acid	1.66	0.003	-0.83	HMDB02545	C08348
33	Iminodiacetic acid	1.86	0.000	0.64	HMDB11753	C19911
34	Methylcitric acid	1.61	0.006	0.42	HMDB00379	
35	N-acetylgalactosamine	1.35	0.037	0.33	HMDB00212	C01074
36	Ornithine-1,5-lactam	1.48	0.017	-0.67	HMDB00323	
37	Phytol	1.85	0.000	1.53	HMDB02019	C01389
38	Threitol	1.33	0.042	-0.81	HMDB04136	C16884

a VIP, Variable Importance in the Projection, was obtained from the OPLS-DA model.
b The *p* value was calculated from univariate statistical analysis.
c FC (fold change) was calculated as a binary logarithm of the average mass response (normalized peak area) ratio between BP vs BPF, where a positive and negative value means that the average mass response of the metabolite in BP is larger and lower than that in BPF, respectively.

Supplementary Table S3 Forty-two metabolites identified by GC-MS in the endosperm-bran fraction with differential content in black point-affected (BP) and black point-free (BPF) kernels

No.	Metabolites	VIP [a]	*p*-value [b]	FC [c]	HMDB	KEGG
1	Succinic acid	1.77	0.0002	-1.18	HMDB00254	C00042
2	Fumaric acid	1.66	0.0017	-1.19	HMDB00134	C00122
3	Malic acid	1.66	0.0017	-1.02	HMDB00156	C00149
4	3-Phosphoglyceric acid	1.56	0.0055	0.75	HMDB00807	C00197
5	Gluconic acid	1.60	0.0037	1.26	HMDB00625	C00257
6	Alanine	1.40	0.0194	0.67	HMDB00161	C00041
7	Aspartic acid	1.72	0.0007	-1.17	HMDB00191	C00049
8	Glutamine	1.59	0.0040	1.4	HMDB00641	C00064
9	Glucaric acid	1.26	0.0441	-1.3	HMDB00663	C00818
10	Threonic acid	1.34	0.0284	-0.75	HMDB00943	C01620
11	Pantothenic acid	1.59	0.0038	-0.97	HMDB00210	C00864
12	Sedoheptulose	1.29	0.0377	-0.45	HMDB03219	C02076
13	2-Aminobutyric acid	1.28	0.0398	0.95	HMDB00452	C02356
14	Cystine	1.32	0.0311	-0.89	HMDB00192	C00491
15	α-Linolenic acid	1.44	0.0141	0.3	HMDB01388	C06427
16	Oleic acid	1.49	0.0097	0.53	HMDB00207	C00712
17	Mannitol	1.45	0.0130	0.81	HMDB00765	C00392
18	Myo-inositol	1.34	0.0274	0.59	HMDB00211	C00137
19	Pyroglutamic acid	1.59	0.0041	0.86	HMDB00267	C01879
20	Glycine	1.28	0.0393	0.53	HMDB00123	C00037
21	Glycolic acid	1.23	0.0491	-0.4	HMDB00115	C00160
22	2-Aminoadipic acid	1.47	0.0114	-0.65	HMDB00510	C00956
23	Nicotinic acid	1.25	0.0458	-0.41	HMDB01488	C00253
24	Arabitol	1.62	0.0029	1.05	HMDB00568	C01904
25	Glucuronic acid	1.33	0.0292	-0.69	HMDB00127	C00191
26	Quinic acid	1.76	0.0003	-2.08	HMDB03072	C00296
27	Allantoin	1.38	0.0214	-0.96	HMDB00462	C01551
28	Guanosine	1.34	0.0281	1.12	HMDB00133	C00387
29	Uric acid	1.56	0.0051	-2.33	HMDB00289	C00366
30	Glutamic acid	1.41	0.0177	-0.63	HMDB00148	C00025
31	Tryptophan	1.24	0.0487	-1.37	HMDB00929	C00078
32	p-Coumaric acid	1.44	0.0140	1.59	HMDB02035	C00811
33	2,4-Dihydroxybutyric acid	1.38	0.0218	-1.11	HMDB00360	
34	Asparagine	1.29	0.0367	-0.63		
35	Erythronic acid	1.32	0.0316	-0.5	HMDB00613	
36	Galacturonic acid	1.54	0.0067	-0.93	HMDB02545	C08348
37	Gycerol-2-phosphate	1.29	0.0369	-0.4		
38	Iminodiacetic acid	1.42	0.0165	-0.66	HMDB11753	C19911
39	Malic acid-1-methylester	1.38	0.0221	-0.92		
40	Methylsuccinic acid	1.47	0.0115	-1.08	HMDB01844	C08645
41	N-acetylgalactosamine	1.33	0.0292	-0.65	HMDB00212	C01074
42	Proline	1.29	0.0366	1.08		

a VIP, Variable Importance in the Projection, was obtained from the OPLS-DA model.
b The *p* value was calculated from univariate statistical analysis.
c FC (fold change) was calculated as a binary logarithm of the average mass response (normalized peak area) ratio between BP vs BPF, where a positive and negative value means that the average mass response of the metabolite in BP is larger and lower than that in BPF, respectively.

Supplementary Table S4 Seventy-three metabolites identified by LC-MS in the germ fraction with differential content between black point-affected (BP) and black point-free (BPF) kernels

No.	Metabolites [a]	RT (min) [b]	m/z [c]	Adduct	ESI mode	VIP [d]	*p*-value [e]	FC [f]
1	DG 36:4; DG(18:2/18:2/0:0)	15.77	634.54	$[M+NH_4]^+$	Pos	5.53	0.0297	-1.76
2	MGDG 36:6; MGDG(18:3/18:3)	16.97	792.56	$[M+NH_4]^+$	Pos	4.82	0.0009	-1.19
3	LysoPC 18:2; PC(18:2/0:0)	10.31	520.34	$[M+H]^+$	Pos	12.89	0.0091	-0.22
4	LysoPE 18:2; PE(18:2/0:0) peak1	10.07	478.29	$[M+H]^+$	Pos	3.67	0.0336	-0.3
5	LysoPE 18:2; PE(18:2/0:0) peak2	10.25	478.29	$[M+H]^+$	Pos	4.93	0.0024	-0.22
6	PA 35:2; PA(17:0/18:2)	13.34	685.48	$[M-H]^-$	Neg	3.36	0.0282	-0.27
7	2-Methoxyhydroquinone	3.58	141.05	$[M+H]^+$	Pos	1.72	0.0005	-0.91
8	Linoleoyl Ethanolamide (LEA)	12.35	324.29	$[M+H]^+$	Pos	7.24	0.0005	-0.8
9	Glutathione	0.91	308.09	$[M+H]^+$	Pos	5.96	0.0249	-0.8
10	Choline	0.81	104.11	$[M+H]^+$	Pos	15.06	0.0122	-0.79
11	Phosphocholine	0.81	184.07	$[M+H]^+$	Pos	2.17	0.0219	-0.75
12	Hexose 6-phosphate	0.73	259.02	$[M-H]^-$	Neg	1.51	0.0216	-0.74
13	Indole	4.48	118.06	$[M+H]^+$	Pos	1.18	0.0273	-0.73
14	1-Benzylimidazole	4.48	159.09	$[M+H]^+$	Pos	1.18	0.0371	-0.7
15	Tryptophan	4.48	205.10	$[M+H]^+$	Pos	5.88	0.0492	-0.7
16	Adenosine	3.31	268.10	$[M+H]^+$	Pos	8.47	0.0213	-0.64
17	Guanine	3.43	152.06	$[M+H]^+$	Pos	1.34	0.0350	-0.38
18	Arginine	0.79	175.12	$[M+H]^+$	Pos	11.88	0.0010	-0.63
19	Trisaccharide	0.9	503.16	$[M-H]^-$	Neg	5.76	0.0011	-0.52
20	Quinic acid	0.8	191.05	$[M-H]^-$	Neg	1.13	0.0340	-0.44
21	Oleamide	13.68	282.28	$[M+H]^+$	Pos	31.36	0.0018	-0.4
22	Homoproline	0.72	130.08	$[M+H]^+$	Pos	1.08	0.0111	-0.38
23	Hydroxyhexadecanoic acid	10.52	271.23	$[M-H]^-$	Neg	5.24	0.0109	0.6
24	Hydroxypentadecanoic acid	9.9	257.21	$[M-H]^-$	Neg	2.31	0.0003	0.92
25	Citric acid	2.11	193.03	$[M+H]^+$	Pos	3.62	0.0206	1.05
26	Trans-Aconitic acid	0.7	173.01	$[M-H]^-$	Neg	2.23	0.0068	0.74
27	DGDG 34:2; DGDG(8:0/26:2)	16.18	934.64	$[M+NH_4]^+$	Pos	10.31	0.0000	2.02
28	FA(C18:2)	12.96	279.23	$[M-H]^-$	Neg	5.69	0.0173	0.91
29	FA(C18:3) peak1	12.24	277.22	$[M-H]^-$	Neg	2.58	0.0027	0.64
30	FA(C18:3) peak2	12.43	277.22	$[M-H]^-$	Neg	2.83	0.0019	0.98
31	FA(C18:3) peak3	13.2	277.22	$[M-H]^-$	Neg	9.14	0.0108	1.61
32	FA(C18:4)	12.61	275.20	$[M-H]^-$	Neg	12.57	0.0425	1.1
33	Ferulic acid	4.65	193.05	$[M-H]^-$	Neg	1.17	0.0186	0.59
34	GlcCer(d14:1/20:0(2OH)) peak 1	13.93	716.56	$[M+H]^+$	Pos	4.74	0.0381	1.04
35	GlcCer(d14:1/20:0(2OH)) peak 2	14.25	716.56	$[M+H]^+$	Pos	3.88	0.0164	1.46
36	DIBOA-hex-hex	4.34	504.13	$[M-H]^-$	Neg	19.78	0.0017	0.69
37	HMBOA-hex-hex	4.58	518.15	$[M-H]^-$	Neg	4.71	0.0214	0.99
38	DIMBOA-hex-hex	4.61	534.14	$[M-H]^-$	Neg	20.98	0.0024	1.71
39	HDMBOA-hex-hex	5.07	548.16	$[M-H]^-$	Neg	2.1	0.0198	3.74
40	Histamine	0.73	112.09	$[M+H]^+$	Pos	1.19	0.0208	1.65
41	Glutamine	0.81	147.07	$[M+H]^+$	Pos	2.28	0.0062	0.96
42	LysoPE 22:3; PE(22:3/0:0)	10.7	534.34	$[M-H]^-$	Neg	3.72	0.0014	0.29
43	MGDG 36:4; MGDG(18:2/18:2)	17.04	796.59	$[M+NH_4]^+$	Pos	17.27	0.0000	1.77
44	MGDG 36:5; MGDG(18:2/18:3)	14.48	794.57	$[M+NH_4]^+$	Pos	5.45	0.0120	1.13
45	N1,N10-Caffeoylferuloylspermidine	5.4	484.24	$[M+H]^+$	Pos	2.46	0.0011	2.96
46	N1,N10-Dicaffeoylspermidine	5.11	470.23	$[M+H]^+$	Pos	1.34	0.0059	1.54
47	N1,N10-Diferuloylspermidine	5.71	498.26	$[M+H]^+$	Pos	12.16	0.0002	2.84
48	N1,N10-Di-p-coumaroylspermidine	5.61	438.24	$[M+H]^+$	Pos	2.02	0.0001	2.72
49	N1,N10-p-Coumaroylferuloylspermidine	5.66	468.25	$[M+H]^+$	Pos	9.54	0.0000	2.83
50	N-Feruloylspermidine	4.05	322.21	$[M+H]^+$	Pos	6.09	0.0000	2.15

Continued Table S4

No.	Metabolites [a]	RT (min) [b]	m/z [c]	Adduct	ESI mode	VIP [d]	*p*-value [e]	FC [f]
51	N-p-Coumaroyl-2-hydroxyputrescine	4.24	251.14	$[M+H]^+$	Pos	3.41	0.0021	2.31
52	N-p-Coumaroylputrescine	4.39	235.14	$[M+H]^+$	Pos	1.11	0.0012	1.07
53	N-p-Coumaroylspermidine	3.55	292.20	$[M+H]^+$	Pos	1.99	0.0321	0.6
54	N-Sinapoylagmatine	4.91	337.19	$[M+H]^+$	Pos	4.27	0.0032	0.33
55	PA 36:6; PA(18:3/18:3)	12.66	691.43	[M-H]-	Neg	3.99	0.0002	0.87
56	PC 34:2; PC(18:2/16:0)	17.36	780.55	$[M+Na]^+$	Pos	4.78	0.0242	1.45
57	p-Coumaric acid	4.34	163.02	$[M-H]^-$	Neg	1.26	0.0450	0.65
58	Jasmonic acid	6.38	209.12	$[M-H]^-$	Neg	1.24	0.0155	0.67
59	Iditol	0.84	181.07	$[M-H]^-$	Neg	5.25	0.0076	0.76
60	Pentitol	0.85	151.06	$[M-H]^-$	Neg	3.21	0.0053	0.66
61	Pentose	0.82	149.04	$[M-H]^-$	Neg	1.04	0.0023	0.49
62	PG 32:0; PG(16:0/16:0)	13.56	721.50	$[M-H]^-$	Neg	2.87	0.0275	0.53
63	PG 32:1; PG(16:0/16:1)	13.2	719.48	$[M-H]^-$	Neg	1.69	0.0349	0.63
64	PG 33:2; PG(15:0/18:2)	13.11	731.48	$[M-H]^-$	Neg	1.51	0.0168	0.55
65	PG 34:2; PG(16:0/18:2)	13.26	745.50	$[M-H]^-$	Neg	19.17	0.0306	0.41
66	PG 34:3; PG(16:0/18:3)	13.06	743.48	$[M-H]^-$	Neg	12.94	0.0075	0.81
67	PI 36:5; PI(18:2/18:3)	12.55	855.50	$[M-H]^-$	Neg	4.5	0.0132	0.35
68	PI 36:6; PI(18:3/18:3)	12.25	853.48	$[M-H]^-$	Neg	2.68	0.0058	1.05
69	PS 34:2; PS(16:0/18:2)	13.16	758.49	$[M-H]^-$	Neg	1.78	0.0156	0.53
70	PS 36:4; PS(18:2/18:2)	12.87	782.49	$[M-H]^-$	Neg	2.16	0.0016	0.58
71	PS 36:5; PS(18:2/18:3)	12.59	780.48	$[M-H]^-$	Neg	2.07	0.0003	0.87
72	Phytosphingosine	9.4	318.30	$[M+H]^+$	Pos	3.77	0.0013	0.48
73	Sphinganine	10.05	302.30	$[M+H]^+$	Pos	2.34	0.0033	0.85

a DG, Diacyl glycerol; DGDG, Digalactosyl diacylglycerol; DGTS, Diacylglyceryltrimethylhomoserine; FA, Fatty acid; Hex, Hexose Glycoside; GlcCer, Glucosylceramide; lysoPC, Lysophosphatidylcholine; lysoPE, Lysophosphatidylethanolamine; MGDG, Monogalactosyl diacylglycerol; PA, Phosphatidic acid; PC, Phosphatidylcholine; PE, Phosphatidylethanolamine; PG, Phosphatidylglycerol; PI, Phosphatidylinositol; PS, Phosphatidylserine.

b RT, retention time (in minute) of chromatographic separation of the compound.

c m/z, mass to charge of the compound with the corresponding adduct.

d VIP, Variable Importance in the Projection, was obtained from the OPLS-DA model.

e The *p* value was calculated from univariate statistical analysis.

f FC (fold change) was calculated as a binary logarithm of the average mass response (normalized peak area) ratio between BP vs BPF, where a positive and negative value means that the average mass response of the metabolite in BP is larger and lower than that in BPF, respectively.

Supplementary Table S5 Sixty-three metabolites identified by LC-MS in the endosperm-bran fraction with differential content between black point-affected (BP) and black point-free (BPF) kernels

No.	Metabolites [a]	RT (min) [b]	m/z [c]	Adduct	ESI mode	VIP [d]	*p*-value [e]	FC [f]
1	DGDG 34:2; DGDG(8:0/26:2)	16.18	934.6409	$[M+NH_4]^+$	Pos	9.56	0.0002	-2.11
2	GlcCer(d14:1/20:0(2OH)) peak 1	13.93	716.562	$[M+H]^+$	Pos	6.23	0.0062	-1.47
3	GlcCer(d14:1/20:0(2OH)) peak 2	14.25	716.5624	$[M+H]^+$	Pos	4.59	0.0045	-1.81
4	MGDG 17:2; MGDG (14:1/3:1)	11.28	534.3616	$[M+NH_4]^+$	Pos	4.48	0.0302	-0.68
5	MGDG 36:5; MGDG(18:2/18:3)	14.48	794.5731	$[M+NH_4]^+$	Pos	6.47	0.0054	-1.56
6	MGDG 36:4; MGDG(18:2/18:2)	17.04	796.5896	$[M+NH_4]^+$	Pos	16.07	0.0007	-1.81
7	LysoPC 18:2; PC(18:2/0:0)	10.31	520.3379	$[M+H]^+$	Pos	13.78	0.0425	-0.27
8	LysoPC 16:0; PC(16:0/0:0)	10.75	496.3383	$[M+H]^+$	Pos	17.42	0.0063	-0.36
9	LysoPE 18:2; PE(18:2/0:0) peak1	10.22	476.2757	$[M-H]^-$	Neg	4.33	0.0156	-0.22
10	LysoPE 18:2; PE(18:2/0:0) peak2	10.25	478.291	$[M+H]^+$	Pos	5.26	0.0229	-0.32
11	LysoPE 16:0; PE(16:0/0:0)	10.64	452.2757	$[M-H]^-$	Neg	3.38	0.0077	-0.26
12	LysoPE 22:3; PE(22:3/0:0)	10.7	534.3357	$[M-H]^-$	Neg	6.12	0.0015	-0.4
13	PE 36:5; PE(18:2/18:3)	12.41	736.4902	$[M-H]^-$	Neg	5.16	0.0161	-0.5
14	PE 36:4; PE(18:2/18:2)	12.67	738.5067	$[M-H]^-$	Neg	22.53	0.0111	-0.55
15	PE 34:2; PE(16:0/18:2)	12.9	714.5066	$[M-H]^-$	Neg	20.75	0.0035	-0.87
16	PE 36:3; PE(18:1/18:2)	12.94	740.5215	$[M-H]^-$	Neg	12.65	0.0044	-0.81
17	PE 32:0; PE(16:0/16:0)	13.12	690.5029	$[M-H]^-$	Neg	2.15	0.0072	-1.07
18	LysoDGTS 16:0; DGTS(16:0/0:0)	11.56	474.376	$[M+H]^+$	Pos	2.66	0.0028	-0.86
19	PG 16:0; PG(16:0/0:0)	9.39	483.2705	$[M-H]^-$	Neg	8.08	0.0040	-0.43
20	PG 36:4; PG(18:2/18:2)	12.87	769.4983	$[M-H]^-$	Neg	4.51	0.0001	-0.37
21	PG 33:2; PG(15:0/18:2)	13.11	731.4818	$[M-H]^-$	Neg	1.84	0.0326	-0.43
22	Quinic acid	0.8	191.0538	$[M-H]^-$	Neg	1.49	0.0016	-1.33
23	Betaine	0.85	118.0854	$[M+H]^+$	Pos	20.75	0.0219	-0.57
24	Aspartic acid	0.82	134.0431	$[M+H]^+$	Pos	1.54	0.0049	-1.18
25	Tryptophan	4.15	203.0817	$[M-H]^-$	Neg	14.09	0.0496	-1.76
26	Succinic acid	0.94	117.018	$[M-H]^-$	Neg	2.1	0.0057	-1.17
27	Fumaric acid	0.71	115.0028	$[M-H]^-$	Neg	5.30	0.0035	-0.99
28	Malic acid	0.71	133.0137	$[M-H]^-$	Neg	8.95	0.0029	-0.95
29	Pantothenic acid	3.93	220.1164	$[M+H]^+$	Pos	2.63	0.0063	-0.97
30	Nicotinic acid	1.37	124.0381	$[M+H]^+$	Pos	1.72	0.0354	-0.54
31	Linoleoyl Ethanolamide (LEA)	12.34	322.2737	$[M-H]^-$	Neg	5.43	0.0078	-1.09
32	N-Sinapoylagmatine	4.91	337.1852	$[M+H]^+$	Pos	1.39	0.0235	-0.95
33	S-Adenosyl-L-homocysteine (SAH)	3.55	383.1116	$[M-H]^-$	Neg	1.15	0.0104	-0.86
34	Phytosphingosine	9.4	318.2993	$[M+H]^+$	Pos	6.09	0.0179	-0.53
35	3-Ketosphinganine	9.53	300.2881	$[M+H]^+$	Pos	2.84	0.0291	-0.71
36	Sphinganine	10.05	302.3041	$[M+H]^+$	Pos	6.94	0.0085	-0.79
37	4-Guanidinobutanoic acid	1.38	146.0915	$[M+H]^+$	Pos	2.27	0.0028	-0.7
38	Raffinose	1.39	503.1596	$[M-H]^-$	Neg	5.99	0.0447	-0.7
39	Sucrose	0.93	341.1073	$[M-H]^-$	Neg	13.08	0.0338	-0.4
40	Trigonelline	0.89	138.0536	$[M+H]^+$	Pos	3.03	0.0018	-0.57
41	Ferulic acid	4.65	193.0493	$[M-H]^-$	Neg	1.72	0.0405	0.53
42	Homoproline	0.72	130.0846	$[M+H]^+$	Pos	1.71	0.0406	0.61
43	PG 36:5; PG(18:2/18:3)	12.82	767.4849	$[M-H]^-$	Neg	2.56	0.0011	0.68
44	PG 34:3; PG(16:0/18:3)	13.06	743.4846	$[M-H]^-$	Neg	4.47	0.0011	0.62
45	PA 36:6; PA(18:3/18:3)	12.66	691.4305	$[M-H]^-$	Neg	1.37	0.0050	0.65
46	PA 37:6; PA(18:3/19:3)	12.87	705.4475	$[M-H]^-$	Neg	2.84	0.0008	0.77
47	PA 36:5; PA(18:2/18:3)	12.92	693.4469	$[M-H]^-$	Neg	3.01	0.0120	0.68
48	PA 34:3; PA(16:0/18:3)	13.18	669.4457	$[M-H]^-$	Neg	1.61	0.0371	0.39
49	PI 36:6; PI(18:3/18:3)	12.25	853.484	$[M-H]^-$	Neg	1.26	0.0024	1.45
50	Carnitine	0.87	162.1105	$[M+H]^+$	Pos	1.26	0.0265	0.66

Continued Table S5

No.	Metabolites [a]	RT (min) [b]	m/z [c]	Adduct	ESI mode	VIP [d]	*p*-value [e]	FC [f]
51	Citric acid	0.71	191.0192	$[M-H]^-$	Neg	4.44	0.0184	0.73
52	Pentitol	0.85	151.06	$[M-H]^-$	Neg	4.81	0.0059	1.1
53	Iditol	0.84	181.0709	$[M-H]^-$	Neg	6.87	0.0393	1.02
54	Glutamine	0.81	147.0748	$[M+H]^+$	Pos	4.09	0.0117	1.64
55	p-Coumaric acid	4.34	163.0214	$[M-H]^-$	Neg	5.06	0.0333	1.68
56	N-p-Coumaroylputrescine	4.39	235.1419	$[M+H]^+$	Pos	1.21	0.0323	0.99
57	N1,N10-Diferuloylspermidine	5.71	498.2574	$[M+H]^+$	Pos	4.83	0.0175	1.36
58	N1,N10-Caffeoylferuloylspermidine	5.4	484.2411	$[M+H]^+$	Pos	4.89	0.0121	1.57
59	N1,N10-p-Coumaroylferuloylspermidine	5.66	468.2472	$[M+H]^+$	Pos	9.45	0.0093	1.8
60	N-p-Coumaroylspermidine	3.55	292.2	$[M+H]^+$	Pos	1.35	0.0058	1.99
61	N1,N10-Dicaffeoylspermidine	5.11	470.2252	$[M+H]^+$	Pos	4.28	0.0108	2.27
62	N1,N10-Di-p-coumaroylspermidine	5.61	438.2358	$[M+H]^+$	Pos	5.07	0.0116	2.78
63	N-p-Coumaroyl-2-hydroxyputrescine	4.24	251.1373	$[M+H]^+$	Pos	5.67	0.0333	3.48

a DG, Diacyl glycerol; DGDG, Digalactosyl diacylglycerol; DGTS, Diacylglyceryltrimethylhomoserine; FA, Fatty acid; Hex, Hexose Glycoside; GlcCer, Glucosylceramide; lysoPC, Lysophosphatidylcholine; lysoPE, Lysophosphatidylethanolamine; MGDG, Monogalactosyl diacylglycerol; PA, Phosphatidic acid; PC, Phosphatidylcholine; PE, Phosphatidylethanolamine; PG, Phosphatidylglycerol; PI, Phosphatidylinositol; PS, Phosphatidylserine.

b RT, retention time (in minute) of chromatographic separation of the compound.

c m/z, mass to charge of the compound with the corresponding adduct.

d VIP, Variable Importance in the Projection, was obtained from the OPLS-DA model.

e The *p* value was calculated from univariate statistical analysis.

f FC (fold change) was calculated as a binary logarithm of the average mass response (normalized peak area) ratio between BP vs BPF, where a positive and negative value means that the average mass response of the metabolite in BP is larger and lower than that in BPF, respectively.

Supplementary Table S6 One hundred and nine metabolites identified by combining LC-MS and MC-MS in the germ fraction with differential content between black point-affected and black point-free kernels

No.	Metabolites [a]	VIP [b]	*p*-value [c]	FC [d]	Analysis method [e]
1	DG 36:4; DG(18:2/18:2/0:0)	5.53	0.03	-1.76	LC-MS
2	2,4-Dihydroxybutyric acid	1.72	0.00	-1.41	GC-MS
3	MGDG 36:6; MGDG(18:3/18:3)	4.82	0.00	-1.19	LC-MS
4	2-Methoxyhydroquinone	1.72	0.00	-0.91	LC-MS
5	Galacturonic acid	1.66	0.00	-0.83	GC-MS
6	Putrescine	1.38	0.03	-0.82	GC-MS
7	Threitol	1.33	0.04	-0.81	GC-MS
8	Linoleoyl Ethanolamide (LEA)	7.24	0.00	-0.8	LC-MS
9	Glutathione	5.96	0.02	-0.8	LC-MS
10	Choline	15.06	0.01	-0.79	LC-MS
11	Phosphocholine	2.17	0.02	-0.75	LC-MS
12	Hexose 6-phosphate	1.51	0.02	-0.74	LC-MS
13	Indole	1.18	0.03	-0.73	LC-MS
14	Uric acid	1.56	0.01	-0.72	GC-MS
15	Myo-inositol-1-phosphate	1.32	0.04	-0.71	GC-MS
16	1-Benzylimidazole	1.18	0.04	-0.7	LC-MS
17	Tryptophan	5.88	0.0492	-0.7	LC-MS
18	Sorbitol	1.41	0.03	-0.68	GC-MS
19	Ornithine-1,5-lactam	1.48	0.02	-0.67	GC-MS
20	Adenosine	8.47	0.02	-0.64	LC-MS
21	Arginine	11.88	0.00	-0.63	LC-MS
22	Threonic acid	1.55	0.01	-0.62	GC-MS
23	Phosphoethanolamine	1.29	0.05	-0.61	GC-MS
24	Ornithine	1.63	0.00	-0.6	GC-MS
25	Glucuronic acid	1.5	0.01	-0.56	GC-MS
26	Trisaccharide	5.76	0.00	-0.52	LC-MS
27	Quinic acid	1.13	0.03	-0.44	LC-MS
28	ADMA(asymmetric dimethylarginine)	1.52	0.01	-0.42	GC-MS
29	5-Methyluridine	1.35	0.04	-0.41	GC-MS
30	Glycine	1.43	0.02	-0.4	GC-MS
31	Oleamide	31.36	0.00	-0.4	LC-MS
32	Guanine	1.34	0.04	-0.38	LC-MS
33	Homoproline	1.08	0.01	-0.38	LC-MS
34	Hypoxanthine	1.37	0.03	-0.32	GC-MS
35	LysoPE 18:2; PE(18:2/0:0) peak1	3.67	0.03	-0.3	LC-MS
36	PA 35:2; PA(17:0/18:2)	3.36	0.03	-0.27	LC-MS
37	LysoPC 18:2; PC(18:2/0:0)	12.89	0.01	-0.22	LC-MS
38	LysoPE 18:2; PE(18:2/0:0) peak2	4.93	0.00	-0.22	LC-MS
39	Myo-inositol	1.55	0.01	-0.21	GC-MS
40	LysoPE 22:3; PE(22:3/0:0)	3.72	0.00	0.29	LC-MS
41	Valine	1.35	0.04	0.31	GC-MS
42	N-acetylgalactosamine	1.35	0.04	0.33	GC-MS
43	N-Sinapoylagmatine	4.27	0.00	0.33	LC-MS
44	PI 36:5; PI(18:2/18:3)	4.5	0.01	0.35	LC-MS
45	Glucose	1.43	0.02	0.37	GC-MS
46	Pantothenic acid	1.58	0.01	0.38	GC-MS
47	PG 34:2; PG(16:0/18:2)	19.17	0.03	0.41	LC-MS
48	Methylcitric acid	1.61	0.01	0.42	GC-MS
49	Isoleucine	1.32	0.04	0.46	GC-MS
50	Arabitol	1.75	0.00	0.48	GC-MS
51	Phytosphingosine	3.77	0.00	0.48	LC-MS

Continued Table S6

No.	Metabolites [a]	VIP [b]	*p*-value [c]	FC [d]	Analysis method [e]
52	Pentose	1.04	0.00	0.49	LC-MS
53	Homocystine	1.82	0.00	0.5	GC-MS
54	Pipecolinic acid	1.53	0.01	0.53	GC-MS
55	PG 32:0; PG(16:0/16:0)	2.87	0.03	0.53	LC-MS
56	PS 34:2; PS(16:0/18:2)	1.78	0.02	0.53	LC-MS
57	2-amino-2-metyl-propanoic acid	1.48	0.02	0.54	GC-MS
58	Uracil	1.39	0.03	0.55	GC-MS
59	PG 33:2; PG(15:0/18:2)	1.51	0.02	0.55	LC-MS
60	PS 36:4; PS(18:2/18:2)	2.16	0.00	0.58	LC-MS
61	Mannitol	1.75	0.00	0.59	GC-MS
62	Ferulic acid	1.17	0.02	0.59	LC-MS
63	Hydroxyhexadecanoic acid	5.24	0.01	0.60	LC-MS
64	N-p-Coumaroylspermidine	1.99	0.03	0.60	LC-MS
65	Citric acid	1.89 (3.62)	0.0000 (0.0206)	0.61 (1.05)	GC-MS (LC-MS)
66	PG 32:1; PG(16:0/16:1)	1.69	0.03	0.63	LC-MS
67	Iminodiacetic acid	1.86	0.00	0.64	GC-MS
68	FA(C18:3) peak1	2.58	0.00	0.64	LC-MS
69	Palmitoleic acid	1.6	0.01	0.65	GC-MS
70	p-Coumaric acid	1.26	0.05	0.65	LC-MS
71	Pentitol	3.21	0.01	0.66	LC-MS
72	Jasmonic acid	1.24	0.02	0.67	LC-MS
73	DIBOA-hex-hex	19.78	0.00	0.69	LC-MS
74	Histidine	1.71	0.00	0.7	GC-MS
75	trans-Aconitic acid	2.23	0.01	0.74	LC-MS
76	Iditol	5.25	0.01	0.76	LC-MS
77	PG 34:3; PG(16:0/18:3)	12.94	0.01	0.81	LC-MS
78	5-Aminovaleric acid	1.84	0.00	0.83	GC-MS
79	2-Aminoadipic acid	1.74	0.00	0.84	GC-MS
80	Sphinganine	2.34	0.00	0.85	LC-MS
81	PA 36:6; PA(18:3/18:3)	3.99	0.00	0.87	LC-MS
82	PS 36:5; PS(18:2/18:3)	2.07	0.00	0.87	LC-MS
83	FA(C18:2)	5.69	0.02	0.91	LC-MS
84	Hydroxypentadecanoic acid	2.31	0.00	0.92	LC-MS
85	FA(C18:3) peak2	2.83	0.00	0.98	LC-MS
86	HMBOA-hex-hex	4.71	0.02	0.99	LC-MS
87	GlcCer(d14:1/20:0(2OH)) peak 1	4.74	0.04	1.04	LC-MS
88	PI 36:6; PI(18:3/18:3)	2.68	0.01	1.05	LC-MS
89	N-p-Coumaroylputrescine	1.11	0.00	1.07	LC-MS
90	FA(C18:4)	12.57	0.04	1.10	LC-MS
91	MGDG 36:5; MGDG(18:2/18:3)	5.45	0.01	1.13	LC-MS
92	Proline	1.43	0.02	1.15	GC-MS
93	Glutamine	1.83 (2.28)	0.0001 (0.0062)	1.18 (0.96)	GC-MS (LC-MS)
94	PC 34:2; PC(18:2/16:0)	4.78	0.02	1.45	LC-MS
95	GlcCer(d14:1/20:0(2OH)) peak 2	3.88	0.02	1.46	LC-MS
96	Phytol	1.85	0.00	1.53	GC-MS
97	N1,N10-Dicaffeoylspermidine	1.34	0.01	1.54	LC-MS
98	FA(C18:3) peak3	9.14	0.01	1.61	LC-MS
99	Histamine	1.19	0.02	1.65	LC-MS
100	DIMBOA-hex-hex	20.98	0.00	1.71	LC-MS
101	MGDG 36:4; MGDG(18:2/18:2)	17.27	0.00	1.77	LC-MS
102	DGDG 34:2; DGDG(8:0/26:2)	10.31	0.00	2.02	LC-MS
103	N-Feruloylspermidine	6.09	0.00	2.15	LC-MS

Continued Table S6

No.	Metabolites [a]	VIP [b]	*p*-value [c]	FC [d]	Analysis method [e]
104	N-p-Coumaroyl-2-hydroxyputrescine	3.41	0.00	2.31	LC-MS
105	N1,N10-Di-p-coumaroylspermidine	2.02	0.00	2.72	LC-MS
106	N1,N10-p-Coumaroylferuloylspermidine	9.54	0.00	2.83	LC-MS
107	N1,N10-Diferuloylspermidine	12.16	0.00	2.84	LC-MS
108	N1,N10-Caffeoylferuloylspermidine	2.46	0.00	2.96	LC-MS
109	HDMBOA-hex-hex	2.10	0.02	3.74	LC-MS

a DG, Diacyl glycerol; DGDG, Digalactosyl diacylglycerol; DGTS, Diacylglyceryltrimethylhomoserine; FA, Fatty acid; Hex, Hexose Glycoside; PA, Phosphatidic acid; GlcCer, Glucosylceramide; lysoPC = Lysophosphatidylcholine; lysoPE, Lysophosphatidylethanolamine; MGDG, Monogalactosyl diacylglycerol; PC, Phosphatidylcholine; PE, Phosphatidylethanolamine; PG, Phosphatidylglycerol; PI, Phosphatidylinositol; PS, Phosphatidylserine

b VIP, variable importance in the projection, was obtained from the OPLS-DA model.

c *p*-value, the p value was calculated from univariate statistical analysis.

d FC (fold change) was calculated as a binary logarithm of the average mass response (normalized peak area) ratio between BP vs BPF, where a positive and negative value means that the average mass response of the metabolite in BP is larger and lower than that in BPF, respectively. BP= black point-affected samples, BPF= black point-free samples.

e GC-MS= Gas Chromatography-Mass Spectrometer, LC-MS= Liquid Chromatograph-Mass Spectrometer. Values of the differential metabolites identify by two-methods were in parentheses.

Supplementary Table S7 Ninety-five metabolites identified by combining LC-MS and MC-MS in the endosperm-bran fraction with differential content between black point-affected (BP) and black point-free (BPF) kernels

No.	Metabolites [a]	VIP [b]	p-value [c]	FC(A/C) [d]	Analysis metnod [e]
1	Succinic acid	1.77(2.10)	0.0002(0.0057)	-1.18(-1.17)	GC-MS
2	Fumaric acid	1.66 (5.30)	0.0017 (0.0035)	-1.19 (-0.99)	GC-MS (LC-MS)
3	Malic acid	1.66 (8.95)	0.0017 (0.0029)	-1.02 (-0.95)	GC-MS (LC-MS)
4	3-Phosphoglyceric acid	1.56	0.0055	0.75	GC-MS
5	Gluconic acid	1.60	0.0037	1.26	GC-MS
6	Alanine	1.40	0.0194	0.67	GC-MS
7	Aspartic acid	1.72 (1.54)	0.0007 (0.0049)	-1.17 (-1.18)	GC-MS (LC-MS)
8	Glutamine	1.59 (4.09)	0.0040 (0.0117)	1.40 (1.64)	GC-MS (LC-MS)
9	Glucaric acid	1.26	0.0441	-1.30	GC-MS
10	Threonic acid	1.34	0.0284	-0.75	GC-MS
11	Pantothenic acid	1.59 (2.63)	0.0038 (0.0063)	-0.97 (-0.97)	GC-MS (LC-MS)
12	Sedoheptulose	1.29	0.0377	-0.45	GC-MS
13	2-Aminobutyric acid	1.28	0.0398	0.95	GC-MS
14	Cystine	1.32	0.0311	-0.89	GC-MS
15	α-Linolenic acid	1.44	0.0141	0.30	GC-MS
16	Oleic acid	1.49	0.0097	0.53	GC-MS
17	Mannitol	1.45	0.0130	0.81	GC-MS
18	Myo-inositol	1.34	0.0274	0.59	GC-MS
19	Pyroglutamic acid	1.59	0.0041	0.86	GC-MS
20	Glycine	1.28	0.0393	0.53	GC-MS
21	Glycolic acid	1.23	0.0491	-0.40	GC-MS
22	2-Aminoadipic acid	1.47	0.0114	-0.65	GC-MS
23	Nicotinic acid	1.25 (1.72)	0.0458 (0.0354)	-0.41 (-0.54)	GC-MS (LC-MS)
24	Arabitol	1.62	0.0029	1.05	GC-MS
25	Glucuronic acid	1.33	0.0292	-0.69	GC-MS
26	Quinic acid	1.76 (1.49)	0.0003 (0.0016)	-2.08 (-1.33)	GC-MS (LC-MS)
27	Allantoin	1.38	0.0214	-0.96	GC-MS
28	Guanosine	1.34	0.0281	1.12	GC-MS
29	Uric acid	1.56	0.0051	-2.33	GC-MS
30	Glutamic acid	1.41	0.0177	-0.63	GC-MS
31	Tryptophan	1.24 (14.09)	0.0487 (0.0496)	-1.37 (-1.76)	GC-MS (LC-MS)
32	p-Coumaric acid	1.44 (5.06)	0.0140 (0.0333)	1.59 (1.68)	GC-MS (LC-MS)
33	2,4-Dihydroxybutyric acid	1.38	0.0218	-1.11	GC-MS
34	Asparagine	1.29	0.0367	-0.63	GC-MS
35	Erythronic acid	1.32	0.0316	-0.5	GC-MS
36	Galacturonic acid	1.54	0.0067	-0.93	GC-MS
37	Gycerol-2-phosphate	1.29	0.0369	-0.40	GC-MS
38	Iminodiacetic acid	1.42	0.0165	-0.66	GC-MS
39	Malic acid-1-methylester	1.38	0.0221	-0.92	GC-MS
40	Methylsuccinic acid	1.47	0.0115	-1.08	GC-MS
41	N-acetylgalactosamine	1.33	0.0292	-0.65	GC-MS
42	Proline	1.29	0.0366	1.08	GC-MS
43	DGDG 34:2; DGDG(8:0/26:2)	9.56	0.0002	-2.11	LC-MS
44	GlcCer(d14:1/20:0(2OH)) peak 1	6.23	0.0062	-1.47	LC-MS
45	GlcCer(d14:1/20:0(2OH)) peak 2	4.59	0.0045	-1.81	LC-MS
46	MGDG 17:2; MGDG (14:1/3:1)	4.48	0.0302	-0.68	LC-MS
47	MGDG 36:5; MGDG(18:2/18:3)	6.47	0.0054	-1.56	LC-MS
48	MGDG 36:4; MGDG(18:2/18:2)	16.07	0.0007	-1.81	LC-MS
49	LysoPC 18:2; PC(18:2/0:0)	13.78	0.0425	-0.27	LC-MS
50	LysoPC 16:0; PC(16:0/0:0)	17.42	0.0063	-0.36	LC-MS
51	LysoPE 18:2; PE(18:2/0:0) peak1	4.33	0.0156	-0.22	LC-MS
52	LysoPE 18:2; PE(18:2/0:0) peak2	5.26	0.0229	-0.32	LC-MS
53	LysoPE 16:0; PE(16:0/0:0)	3.38	0.0077	-0.26	LC-MS
54	LysoPE 22:3; PE(22:3/0:0)	6.12	0.0015	-0.4	LC-MS
55	PE 36:5; PE(18:2/18:3)	5.16	0.0161	-0.5	LC-MS
56	PE 36:4; PE(18:2/18:2)	22.53	0.0111	-0.55	LC-MS

Continued Table S7

No.	Metabolites [a]	VIP [b]	p-value [c]	FC(A/C) [d]	Analysis metnod [e]
57	PE 34:2; PE(16:0/18:2)	20.75	0.0035	-0.87	LC-MS
58	PE 36:3; PE(18:1/18:2)	12.65	0.0044	-0.81	LC-MS
59	PE 32:0; PE(16:0/16:0)	2.15	0.0072	-1.07	LC-MS
60	lysoDGTS 16:0; DGTS(16:0/0:0)	2.66	0.0028	-0.86	LC-MS
61	PG 16:0; PG(16:0/0:0)	8.08	0.0040	-0.43	LC-MS
62	PG 36:4; PG(18:2/18:2)	4.51	0.0001	-0.37	LC-MS
63	PG 33:2; PG(15:0/18:2)	1.84	0.0326	-0.43	LC-MS
64	Betaine	20.75	0.0219	-0.57	LC-MS
65	Linoleoyl Ethanolamide (LEA)	5.43	0.0078	-1.09	LC-MS
66	N-Sinapoylagmatine	1.39	0.0235	-0.95	LC-MS
67	S-Adenosyl-L-homocysteine (SAH)	1.15	0.0104	-0.86	LC-MS
68	Phytosphingosine	6.09	0.0179	-0.53	LC-MS
69	3-Ketosphinganine	2.84	0.0291	-0.71	LC-MS
70	Sphinganine	6.94	0.0085	-0.79	LC-MS
71	4-Guanidinobutanoic acid	2.27	0.0028	-0.7	LC-MS
72	Raffinose	5.99	0.0447	-0.7	LC-MS
73	Sucrose	13.08	0.0338	-0.4	LC-MS
74	Trigonelline	3.03	0.0018	-0.57	LC-MS
75	Ferulic acid	1.72	0.0405	0.53	LC-MS
76	Homoproline	1.71	0.0406	0.61	LC-MS
77	PG 36:5; PG(18:2/18:3)	2.56	0.0011	0.68	LC-MS
78	PG 34:3; PG(16:0/18:3)	4.47	0.0011	0.62	LC-MS
79	PA 36:6; PA(18:3/18:3)	1.37	0.0050	0.65	LC-MS
80	PA 37:6; PA(18:3/19:3)	2.84	0.0008	0.77	LC-MS
81	PA 36:5; PA(18:2/18:3)	3.01	0.0120	0.68	LC-MS
82	PA 34:3; PA(16:0/18:3)	1.61	0.0371	0.39	LC-MS
83	PI 36:6; PI(18:3/18:3)	1.26	0.0024	1.45	LC-MS
84	Carnitine	1.26	0.0265	0.66	LC-MS
85	Citric acid	4.44	0.0184	0.73	LC-MS
86	Pentitol	4.81	0.0059	1.1	LC-MS
87	Iditol	6.87	0.0393	1.02	LC-MS
88	N-p-Coumaroylputrescine	1.21	0.0323	0.99	LC-MS
89	N1,N10-Diferuloylspermidine	4.83	0.0175	1.36	LC-MS
90	N1,N10-Caffeoylferuloylspermidine	4.89	0.0121	1.57	LC-MS
91	N1,N10-p-Coumaroylferuloylspermidine	9.45	0.0093	1.8	LC-MS
92	N-p-Coumaroylspermidine	1.35	0.0058	1.99	LC-MS
93	N1,N10-Dicaffeoylspermidine	4.28	0.0108	2.27	LC-MS
94	N1,N10-Di-p-coumaroylspermidine	5.07	0.0116	2.78	LC-MS
95	N-p-Coumaroyl-2-hydroxyputrescine	5.67	0.0333	3.48	LC-MS

a DG, Diacyl glycerol; DGDG, Digalactosyl diacylglycerol; DGTS, Diacylglyceryltrimethylhomoserine; FA, Fatty acid; Hex, Hexose Glycoside; PA, Phosphatidic acid; GlcCer, Glucosylceramide; lysoPC = Lysophosphatidylcholine; lysoPE, Lysophosphatidylethanolamine; MGDG, Monogalactosyl diacylglycerol; PC, Phosphatidylcholine; PE, Phosphatidylethanolamine; PG, Phosphatidylglycerol; PI, Phosphatidylinositol; PS, Phosphatidylserine

b VIP, variable importance in the projection, was obtained from the OPLS-DA model.

c *p*-value, the p value was calculated from univariate statistical analysis.

d FC (fold change) was calculated as a binary logarithm of the average mass response (normalized peak area) ratio between BP vs BPF, where a positive and negative value means that the average mass response of the metabolite in BP is larger and lower than that in BPF, respectively.

e GC-MS= Gas Chromatography-Mass Spectrometer, LC-MS= Liquid Chromatograph-Mass Spectrometer. Values of the differential metabolites identify by two-methods were in parentheses.

Supplementary Table S8 One hundred and fifty-six metabolites identified by combining LC-MS and MC-MS with differential content between black point-affected (BP) and black point-free (BPF) kernels

No.	Metabolites	Germ fraction	Endosperm-bran fraction	Method [a]
1	1-Benzylimidazole	-0.7		LC-MS
2	2,4-dihydroxybutyric acid	-1.41	-1.11	GC-MS
3	2-amino-2-metyl-propanoic acid	0.54		GC-MS
4	2-Aminoadipic acid	0.84	-0.65	GC-MS
5	2-Aminobutyric acid	0.95		GC-MS
6	2-Methoxyhydroquinone	-0.91		LC-MS
7	3-Ketosphinganine		-0.71	LC-MS
8	3-phosphoglyceric acid		0.75	GC-MS
9	4-Guanidinobutanoic acid		-0.7	LC-MS
10	5-aminovaleric acid	0.83		GC-MS
11	5-methyluridine	-0.41		GC-MS
12	Adenosine	-0.64		LC-MS
13	ADMA(asymmetric dimethylarginine)	-0.42		GC-MS
14	Alanine		0.67	GC-MS
15	Allantoin		-0.96	GC-MS
16	Arabitol	0.48	1.05	GC-MS
17	Arginine	-0.63		LC-MS
18	Asparagine		-0.63	GC-MS
19	Aspartic acid		-1.17 (-1.18)	GC-MS (LC-MS)
20	Betaine		-0.57	LC-MS
21	Carnitine		0.66	LC-MS
22	Choline	-0.79		LC-MS
23	Citric acid	0.61 (1.05)	0.73	GC-MS (LC-MS)
24	Cystine		-0.89	GC-MS
25	DG 36:4; DG(18:2/18:2/0:0)	-1.76		LC-MS
26	DGDG 34:2; DGDG(8:0/26:2)	2.02	-2.11	LC-MS
27	DIBOA-hex-hex	0.69		LC-MS
28	DIMBOA-hex-hex	1.71		LC-MS
29	Erythronic acid		-0.5	GC-MS
30	FA(C18:2)	0.91		LC-MS
31	FA(C18:3) peak1	0.64		LC-MS
32	FA(C18:3) peak2	0.98		LC-MS
33	FA(C18:3) peak3	1.61		LC-MS
34	FA(C18:4)	1.1		LC-MS
35	Ferulic acid	0.59	0.53	LC-MS
36	Fumaric acid		-1.19 (-0.99)	GC-MS (LC-MS)
37	Galacturonic acid	-0.83	-0.93	GC-MS
38	GlcCer(d14:1/20:0(2OH)) peak 1	1.04	-1.47	LC-MS
39	GlcCer(d14:1/20:0(2OH)) peak 2	1.46	-1.81	LC-MS
40	Glucaric acid		-1.30	GC-MS
41	Gluconic acid		1.26	GC-MS
42	Glucose	0.37		GC-MS
43	Glucuronic acid	-0.56	-0.69	GC-MS
44	Glutamic acid		-0.63	GC-MS
45	Glutamine	1.18 (0.96)	1.40 (1.64)	GC-MS (LC-MS)
46	Glutathione	-0.8		LC-MS
47	Glycine	-0.4	0.53	GC-MS
48	Glycolic acid		-0.40	GC-MS
49	Guanine	-0.38		LC-MS
50	Guanosine		1.12	GC-MS
51	Gycerol-2-phosphate		-0.40	GC-MS
52	HDMBOA-hex-hex	3.74		LC-MS
53	Hexose 6-phosphate	-0.74		LC-MS
54	Histamine	1.65		LC-MS
55	Histidine	0.7		GC-MS
56	HMBOA-hex-hex	0.99		LC-MS
57	Homocystine	0.5		GC-MS

Continued Table S8

No.	Metabolites	Germ fraction	Endosperm-bran fraction	Method [a]
58	Homoproline	-0.38	0.61	LC-MS
59	Hydroxyhexadecanoic acid	0.6		LC-MS
60	Hydroxypentadecanoic acid	0.92		LC-MS
61	Hypoxanthine	-0.32		GC-MS
62	Iditol	0.76	1.02	LC-MS
63	Iminodiacetic acid	0.64	-0.66	GC-MS
64	Indole	-0.73		LC-MS
65	Isoleucine	0.46		GC-MS
66	Jasmonic acid	0.67		LC-MS
67	Linoleoyl Ethanolamide (LEA)	-0.8	-1.09	LC-MS
68	lysoDGTS 16:0; DGTS(16:0/0:0)		-0.86	LC-MS
69	lysoPC 16:0; PC(16:0/0:0)		-0.36	LC-MS
70	LysoPC 18:2; PC(18:2/0:0)	-0.22	-0.27	LC-MS
71	lysoPE 16:0; PE(16:0/0:0)		-0.26	LC-MS
72	LysoPE 18:2; PE(18:2/0:0) peak1	-0.3	-0.22	LC-MS
73	LysoPE 18:2; PE(18:2/0:0) peak2	-0.22	-0.32	LC-MS
74	LysoPE 22:3; PE(22:3/0:0)	0.29	-0.4	LC-MS
75	Malic acid		-1.02 (-0.95)	GC-MS (LC-MS)
76	Malic acid-1-methylester		-0.92	GC-MS
77	Mannitol	0.59	0.81	GC-MS
78	Methylcitric acid	0.42		GC-MS
79	Methylsuccinic acid		-1.08	GC-MS
80	MGDG 17:2; MGDG (14:1/3:1)		-0.68	LC-MS
81	MGDG 36:4; MGDG(18:2/18:2)	1.77	-1.81	LC-MS
82	MGDG 36:5; MGDG(18:2/18:3)	1.13	-1.56	LC-MS
83	MGDG 36:6; MGDG(18:3/18:3)	-1.19		LC-MS
84	Myo-inositol	-0.21	0.59	GC-MS
85	Myo-inositol-1-phosphate	-0.71		GC-MS
86	N1,N10-Caffeoylferuloylspermidine	2.96	1.57	LC-MS
87	N1,N10-Dicaffeoylspermidine	1.54	2.27	LC-MS
88	N1,N10-Diferuloylspermidine	2.84	1.36	LC-MS
89	N1,N10-Di-p-coumaroylspermidine	2.72	2.78	LC-MS
90	N1,N10-p-Coumaroylferuloylspermidine	2.83	1.8	LC-MS
91	N-acetylgalactosamine	0.33	-0.65	GC-MS
92	N-Feruloylspermidine	2.15		LC-MS
93	Nicotinic acid		-0.41 (-0.54)	GC-MS (LC-MS)
94	N-p-Coumaroyl-2-hydroxyputrescine	2.31	3.48	LC-MS
95	N-p-Coumaroylputrescine	1.07	0.99	LC-MS
96	N-p-Coumaroylspermidine	0.6	1.99	LC-MS
97	N-Sinapoylagmatine	0.33	-0.95	LC-MS
98	Oleamide	-0.4		LC-MS
99	Oleic acid		0.53	GC-MS
100	Ornithine	-0.6		GC-MS
101	Ornithine-1,5-lactam	-0.67		GC-MS
102	PA 34:3; PA(16:0/18:3)		0.39	LC-MS
103	PA 35:2; PA(17:0/18:2)	-0.27		LC-MS
104	PA 36:5; PA(18:2/18:3)		0.68	LC-MS
105	PA 36:6; PA(18:3/18:3)	0.87	0.65	LC-MS
106	PA 37:6; PA(18:3/19:3)		0.77	LC-MS
107	Palmitoleic acid	0.65		GC-MS
108	Pantothenic acid	0.38	-0.97 (-0.97)	GC-MS (LC-MS)
109	PC 34:2; PC(18:2/16:0)	1.45		LC-MS
110	p-Coumaric acid	0.65	1.59 (1.68)	GC-MS (LC-MS)
111	PE 32:0; PE(16:0/16:0)		-1.07	LC-MS
112	PE 34:2; PE(16:0/18:2)		-0.87	LC-MS
113	PE 36:3; PE(18:1/18:2)		-0.81	LC-MS
114	PE 36:4; PE(18:2/18:2)		-0.55	LC-MS
115	PE 36:5; PE(18:2/18:3)		-0.5	LC-MS
116	Pentitol	0.66	1.1	LC-MS

Continued Table S8

No.	Metabolites	Germ fraction	Endosperm-bran fraction	Method [a]
117	Pentose	0.49		LC-MS
118	PG 16:0; PG(16:0/0:0)		-0.43	LC-MS
119	PG 32:0; PG(16:0/16:0)	0.53		LC-MS
120	PG 32:1; PG(16:0/16:1)	0.63		LC-MS
121	PG 33:2; PG(15:0/18:2)	0.55	-0.43	LC-MS
122	PG 34:2; PG(16:0/18:2)	0.41		LC-MS
123	PG 34:3; PG(16:0/18:3)	0.81	0.62	LC-MS
124	PG 36:4; PG(18:2/18:2)		-0.37	LC-MS
125	PG 36:5; PG(18:2/18:3)		0.68	LC-MS
126	Phosphocholine	-0.75		LC-MS
127	Phosphoethanolamine	-0.61		GC-MS
128	Phytol	1.53		GC-MS
129	Phytosphingosine	0.48	-0.53	LC-MS
130	PI 36:5; PI(18:2/18:3)	0.35		LC-MS
131	PI 36:6; PI(18:3/18:3)	1.05	1.45	LC-MS
132	Pipecolinic acid	0.53		GC-MS
133	Proline	1.15	1.08	GC-MS
134	PS 34:2; PS(16:0/18:2)	0.53		LC-MS
135	PS 36:4; PS(18:2/18:2)	0.58		LC-MS
136	PS 36:5; PS(18:2/18:3)	0.87		LC-MS
137	Putrescine	-0.82		GC-MS
138	Pyroglutamic acid		0.86	GC-MS
139	Quinic acid	-0.44	-2.08 (-1.33)	GC-MS (LC-MS)
140	Raffinose		-0.7	LC-MS
141	S-Adenosyl-L-homocysteine (SAH)		-0.86	LC-MS
142	Sedoheptulose		-0.45	GC-MS
143	Sorbitol	-0.68		GC-MS
144	Sphinganine	0.85	-0.79	LC-MS
145	Succinic acid	-1.18	-1.17	GC-MS (LC-MS)
146	Sucrose		-0.4	LC-MS
147	Threitol	-0.81		GC-MS
148	Threonic acid	-0.62	-0.75	GC-MS
149	trans-Aconitic acid	0.74		LC-MS
150	Trigonelline		-0.57	LC-MS
151	Trisaccharide	-0.52		LC-MS
152	Tryptophan	-0.7	-1.37 (-1.76)	GC-MS (LC-MS)
153	Uracil	0.55		GC-MS
154	Uric acid	-0.72	-2.33	GC-MS
155	Valine	0.31		GC-MS
156	α-Linolenic acid		0.30	GC-MS

a GC-MS= Gas Chromatography-Mass Spectrometer, LC-MS= Liquid Chromatograph-Mass Spectrometer. Values of the differential metabolite
